# Integrative Analysis of MicroRNA and mRNA Data Reveals an Orchestrated Function of MicroRNAs in Skeletal Myocyte Differentiation in Response to TNF-α or IGF1

**DOI:** 10.1371/journal.pone.0135284

**Published:** 2015-08-13

**Authors:** Swanhild U. Meyer, Steffen Sass, Nikola S. Mueller, Stefan Krebs, Stefan Bauersachs, Sebastian Kaiser, Helmut Blum, Christian Thirion, Sabine Krause, Fabian J. Theis, Michael W. Pfaffl

**Affiliations:** 1 Physiology Weihenstephan, Technische Universität München, Freising, Germany; 2 Institute of Computational Biology, Helmholtz Center Munich, German Research Center for Environmental Health, Neuherberg, Germany; 3 Laboratory for Functional Genome Analysis (LAFUGA), Gene Center, Ludwig-Maximilians-Universität München, Munich, Germany; 4 Department of Statistics, Ludwig-Maximilians-Universität München, Munich, Germany; 5 SIRION Biotech GmbH, Martinsried, Germany; 6 Friedrich-Baur-Institute, Department of Neurology, Ludwig-Maximilians-Universität München, Munich, Germany; 7 Department of Mathematics, Technische Universität München, Garching, Germany; University of Massachusetts Medical, UNITED STATES

## Abstract

**Introduction:**

Skeletal muscle cell differentiation is impaired by elevated levels of the inflammatory cytokine tumor necrosis factor-α (TNF-α) with pathological significance in chronic diseases or inherited muscle disorders. Insulin like growth factor-1 (IGF1) positively regulates muscle cell differentiation. Both, TNF-α and IGF1 affect gene and microRNA (miRNA) expression in this process. However, computational prediction of miRNA-mRNA relations is challenged by false positives and targets which might be irrelevant in the respective cellular transcriptome context. Thus, this study is focused on functional information about miRNA affected target transcripts by integrating miRNA and mRNA expression profiling data.

**Methodology/Principal Findings:**

Murine skeletal myocytes PMI28 were differentiated for 24 hours with concomitant TNF-α or IGF1 treatment. Both, mRNA and miRNA expression profiling was performed. The data-driven integration of target prediction and paired mRNA/miRNA expression profiling data revealed that i) the quantity of predicted miRNA-mRNA relations was reduced, ii) miRNA targets with a function in cell cycle and axon guidance were enriched, iii) differential regulation of anti-differentiation miR-155-5p and miR-29b-3p as well as pro-differentiation miR-335-3p, miR-335-5p, miR-322-3p, and miR-322-5p seemed to be of primary importance during skeletal myoblast differentiation compared to the other miRNAs, iv) the abundance of targets and affected biological processes was miRNA specific, and v) subsets of miRNAs may collectively regulate gene expression.

**Conclusions:**

Joint analysis of mRNA and miRNA profiling data increased the process-specificity and quality of predicted relations by statistically selecting miRNA-target interactions. Moreover, this study revealed miRNA-specific predominant biological implications in skeletal muscle cell differentiation and in response to TNF-α or IGF1 treatment. Furthermore, myoblast differentiation-associated miRNAs are suggested to collectively regulate gene clusters and targets associated with enriched specific gene ontology terms or pathways. Predicted miRNA functions of this study provide novel insights into defective regulation at the transcriptomic level during myocyte proliferation and differentiation due to inflammatory stimuli.

## Introduction

Adult skeletal myoblast differentiation is important for muscle repair after injury and involves a multistep process including proliferation, exit from the cell cycle, migration, and cell fusion into multinuclear myotubes [1–3]. Most of the progressive muscle disorders are associated with ineffective or burn-out regenerative potential of muscle tissue [2]. In muscle disorders or other chronic diseases pro-inflammatory cytokines, such as TNF-α are elevated. TNF- α can impair myoblast differentiation [4] by inhibiting the initiation of differentiation [5] as myoblasts could not exit the cell cycle as efficiently as controls [6]. Adversely, IGF1 can facilitate myoblast differentiation at certain concentrations [7,5]. Besides, post-transcriptional regulators such as microRNAs (miRNAs) have been shown to be powerful regulators in the process of skeletal muscle cell differentiation [8–10]. Moreover, many muscular disorders, which involve inflammation and impaired muscle regeneration [11], show miRNA deregulation at various levels [12]. Interestingly, the precise impact of TNF-α or IGF1 on the miRNA and mRNA transcriptome of differentiating skeletal muscle cells remains to be elucidated. We aimed at understanding the impact of TNF-α and IGF1 exposure on predicted miRNA-target interactions of murine skeletal muscle differentiation. As prediction of miRNA-mRNA relations solely based on computational approaches bears high numbers of false positive predictions [13] we proposed the simultaneous interpretation of real experimental expression data together with target prediction. For this approach we assumed that miRNAs mainly inversely regulate mRNAs by promoting mRNA destabilization [14]. In this mode, we assessed results and data interpretations derived from inversely associated miRNA and mRNA expression profiling data of differentiating murine skeletal muscle cells and the effect of TNF- α or IGF1 treatment. We evaluated results from joint miRNA-mRNA analysis by taking into account the number of targets, specifically transcription factors, co-expression of mRNAs and miRNAs, functional enrichment, as well as concerted and redundant target regulation following the guidelines suggested by Meyer et al. [15] with slight modifications and extensions. It had been suggested that coordinated post-transcriptional regulation by miRNAs [16] and cooperativity of miRNA-target interaction was a widespread phenomenon that may play an important role in miRNA-mediated gene regulation [17]. We identified miRNA specific biological implications, gene ontology and pathway enrichments of differentiation-associated miRNAs, as well as regulation of functionally related transcription factors, and indications for a coordinated function of differentiation-associated miRNAs. Moreover, we showed a strategy how to reduce the complexity of possible miRNA-mRNA interactions to predict physiologically relevant associations more accurately.

## Materials and Methods

### Cell culture

The murine skeletal myoblast cell line PMI28 [18] was cultured in a growth medium composed of Ham’s F10 (PAA Laboratories GmbH, Pasching, Austria), supplemented with 20% fetal bovine serum (Sigma-Aldrich, St. Louis, MO, USA), 2 mM L-glutamine (PAA Laboratories), and Penicillin (100 I.U./ml) / Streptomycin (100 μg/ml, PAA Laboratories). 24 hours after seeding of the cells the growth medium was replaced by a differentiation medium containing DMEM medium with 2% horse serum (Gibco, Life Technologies GmbH, Darmstadt, Germany), 2 mM L-glutamine (PAA Laboratories), and Penicillin (100 I.U./ml) / Streptomycin (100 μg/ml) (PAA Laboratories). The differentiation medium of the treatment groups additionally contained 2 x 10^3^ U/ml murine recombinant TNF-α (Roche Diagnostics, Rotkreuz, Switzerland) or 5 ng/ml murine recombinant IGF1 (Sigma-Aldrich). The control and treatment media were replenished twice a day to ensure cytokine and growth factor activity. Murine PMI28 cells were harvested 24 h after the induction of fusion by serum withdrawal for RNA analyses. Cells were propagated and differentiated at 37°C in 80% relative humidity and 5% CO_2_.

### RNA extraction and quality control

About 2 x 10^6^ cells per sample were harvested in 1.5 ml Trizol (Life Technologies GmbH, Darmstadt, Germany), homogenized and mixed with 0.45 ml chloroform. Phases were separated by centrifugation. For RNA precipitation the upper aqueous phase was aspirated and 1.25 ml isopropanol were added, mixed and centrifuged. Subsequently the pellet was washed with 75% ethanol, then dried and finally dissolved in water. The total RNA concentration of individual samples was determined photometrically using the NanoDrop 1000 ND-1000 (Peqlab, Erlangen, Germany). Overall RNA quality was assessed by gel electrophoresis using a 1% agarose gel with a 1 KB molecular weight marker separated in parallel.

### Gene expression profiling by hybridization microarrays

Gene expression profiling was performed for triplicate samples with GeneChip Mouse Gene 1.0 ST Arrays (901169/901171, Affymetrix, Santa Clara, CA, USA) following the manufacturer’s instructions. For cDNA synthesis 250 ng total RNA were used applying the GeneChip Poly-A RNA Control Kit (900433, Affymetrix) and Ambion WT Expression Kit (Ambion, Life Technologies GmbH, Darmstadt, Germany) according to the manufacturer’s instructions. After analysis of cDNA yield and size distribution the purified cDNA was fragmented, labeled and hybridized applying the GeneChip WT Terminal Labeling and Controls Kits (Affymetrix) following the manufacturer’s instructions. Washing and staining was carried out by utilizing the GeneChip Hybridization, Wash, and Stain Kit (Affymetrix) according to the manufacturer’s instructions. The array was scanned and data were acquired with the GACC Scan Control Software. Affymetrix CEL files were read, normalized and summarized using the RMA method [19] as implemented in the Affymetrix apt package. Probe sets were filtered for having at least two ‘detected above background’ present calls in at least one experimental group. GeneChip Mouse Gene 1.0 ST Array data was MIAME [20] compliant and were submitted to the ArrayExpress database (www.ebi.ac.uk/arrayexpress) [21], a publicly available repository consistent with the MIAME guidelines. Data are available with the ArrayExpress accession number E-MTAB-3474.

### miRNA profiling by microarray technology

MiRNA expression profiling by microarray technology was performed using Mouse miRNA Microarray Release 15.0 (8x15K, G4471A-029152, Agilent Technologies, Böblingen, Germany) which contained probes for 696 miRNAs from Sanger miRBase release 15.0. Quadruplicate samples of murine skeletal myoblasts, differentiated myotubes, and TNF-α or IGF1 treated myotubes were profiled. We used 100 ng total RNA for Cy3-labeling of miRNAs by utilizing the miRNA Complete Labeling and Hybridization Kit (Agilent Technologies) according to the manufacturer’s instructions. The samples were hybridized to the microarray at 55°C for 20 hours. Subsequently, the microarrays were washed and scanned with the Agilent Microarray Scanner G2505C in a single pass mode with a scan resolution of 3 μm, 20 bit mode. Preprocessing of data included extraction of signal intensities and background by using Feature Extraction Software 10.7.3.1 (Agilent Technologies). If a miRNA passed the filtering criterion “is well above background” in at least two of the replicates within one group it was retained. MiRNA microarray data was normalized by loessM normalization [22,23]. The Agilent microarray data was MIAME [20] compliant. The miRNA microarray expression data were deposited in the ArrayExpress database [21] and are accessible with the ArrayExpress accession number E-MTAB-1114.

### miRNA profiling by quantitative qPCR

TaqMan Rodent MicroRNA Arrays 2.0 (Life Technologies) were utilized for qPCR based miRNA expression profiling. We performed three separate reverse transcription reactions per sample using 150 ng total RNA each and Megaplex RT Primers Rodent Pool A and Rodent Pool B (Life Technologies) following the manufacturer’s protocol. Subsequently, each reverse transcription reaction was pre-amplified using the Megaplex PreAmp Primers Rodent Pool A and Rodent Pool B (Life Technologies) according to the manufacturer’s instructions. For each biological sample the three separate reverse transcription and preamplification reactions were pooled. The qPCR reaction mix was prepared according to the manufacturer’s instructions. The arrays were run on an Applied Biosystems 7900HT Fast Real-Time System with cycling conditions according to the manufacturer’s protocol. SDS 2.3 software (Life Technologies) was applied for obtaining raw expression data. The SDS files were loaded into the RQ Manager 1.2 software (Applied Biosystems, Life Technologies). Each amplification plot was reviewed manually. The threshold was set to 0.2 and adjusted for individual assays if necessary. We retained miRNAs which showed Cq-values smaller than 32 in at least two of the corresponding triplicates of a group for further data processing. We applied loessM normalization [22,23] using R programming [24].

### Statistics

MiRNA expression profiling data was tested for significant differences by applying significance analysis of microarrays (SAM) [25] which uses permutation. We performed false discovery rate (FDR) correction of p-values. Significance analysis and FDR correction were performed within the R environment for statistical computing [24]. Differentially expressed genes withing the mRNA profiling data set were determined with limma [26] using a factorial design with treatment and time-point as factors. Pairwise comparisons were extracted for all combinations of consecutive time points for the same treatment and between all treatments at the same time-point. Expression profiles of all samples for all probesets that were called significantly different (fdr<0.01 and log2fold change>1) in at least one pairwise comparison were clustered with the self-organizing tree algorithm (SOTA) method [27].

### Bioinformatical analysis of data

We selected 21 miRNAs which were detected on both miRNA platforms, the microarray and the qPCR array, for the joint analysis of miRNA and mRNA profiling data. We used miRNA target predictions based on TargetScan (Release 6.2, June 2012) (http://www.targetscan.org/) [28] and miRanda (release August 2010) (http://www.microrna.org/) [29]. Integrated analysis of the inverse relations of expressed miRNAs and mRNAs in conjunction with target predictions was done as follows: We set up an initial miRNA-mRNA target network by unifying the predicted targets from TargetScan and miRanda. We then used the *glmnet* package [30] for the *R* statistical environment [24] in order to fit a generalized linear model where the expression profiles of the predicted miRNAs served as predictor variables and the mRNA expression profile as response. This regression model was then used to perform a feature selection on the miRNAs utilizing the elastic net penalty [31]. The penalty parameter was determined by 10-fold cross-validation. We furthermore introduced a negativity constraint on the coefficients of the regression model in order to allow only negative effects of the miRNAs on the mRNA. This procedure was applied for miRNA expression measurements derived from both, microarray and qPCR analysis. The intersection network was then defined as the intersection of the miRNA-mRNA relationships from the two platforms. We then applied a local enrichment on the intersection network analysis for gene sets derived from KEGG pathways (http://www.genome.jp/kegg/pathway.html) and self-compiled gene sets. Initially, we defined a gene set for each gene in the network containing all other genes that were targeted by the same miRNA as well as the respective gene itself. We then applied Fisher’s exact test on each of these gene sets to test for statistical significant overrepresentation of genes assigned to a certain KEGG pathway. Hence, we obtained a p-value for each gene indicating the overrepresentation of the pathway gene set in the neighborhood of this gene.

Moreover, we applied Genomatix Pathway System (GePS) within the Genomatix Software Suite (Genomatix, Munich, Germany) to identify and display enriched canonical pathways, gene ontology terms, disease terms, and transcription factors based on information extracted from public and proprietary databases, such as pathway data from the Pathway Interaction Database [32], and co-citation in the literature [33]. For clustering of differential gene expressions we used SOTA [34,35] implemented in the MultiExperiment Viewer (MeV) (http://www.tm4.org/mev.html) with settings to receive nine clusters.

## Results

**Fig 1:**
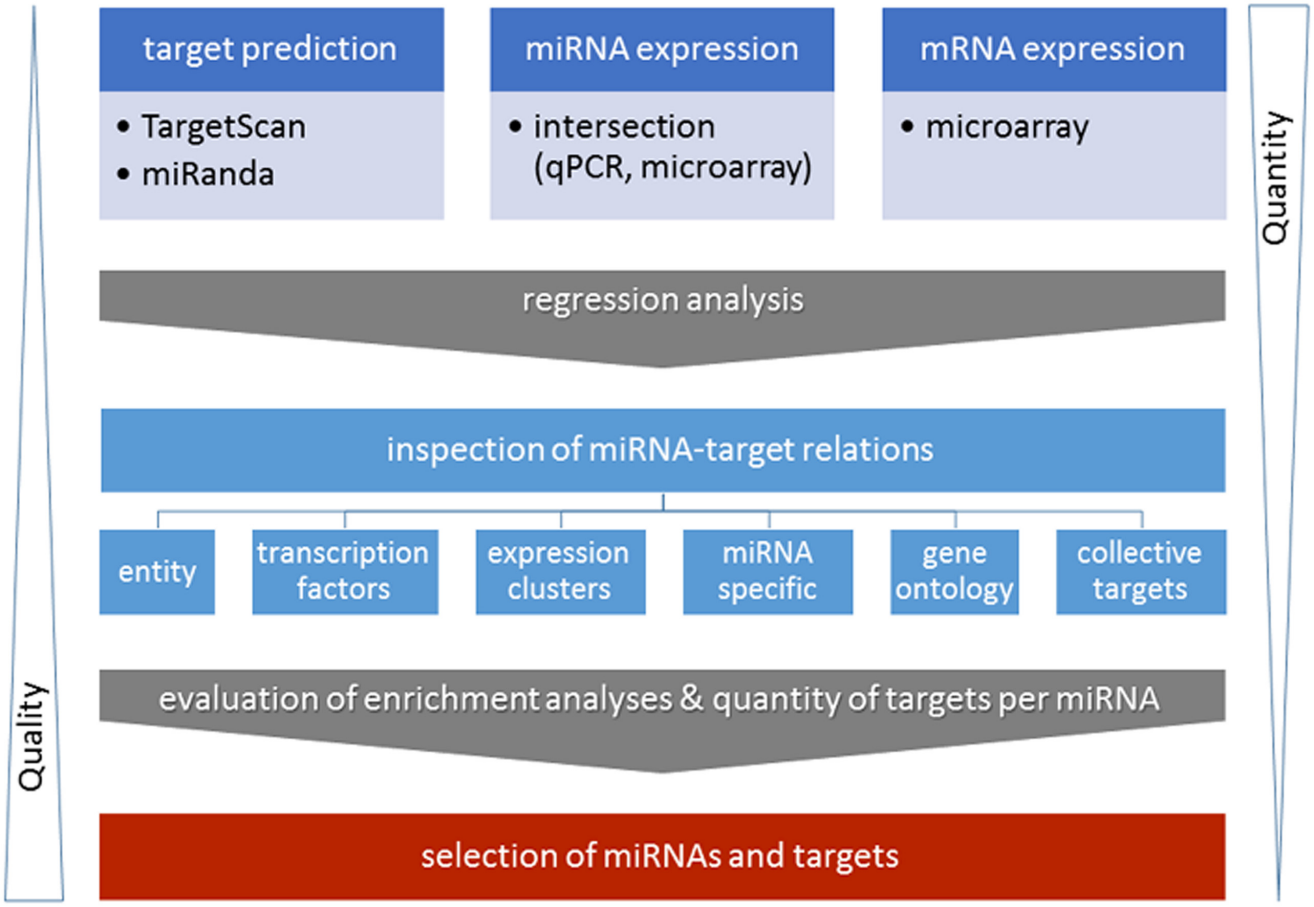
Joint analysis of miRNA and mRNA expression data. Schematic overview of the integrative analysis of miRNA target prediction based on TargetScan (www.targetscan.org/) and miRanda (www.microrna.org/) and paired miRNA/mRNA expression data derived from the same experiments. The graphic is an extension and modification of Fig 1 published by Meyer and co-workers 2014 [15]. The expression data included the intersection dataset derived from miRNA microarray and miRNA qPCR profiling experiments and was inversely associated to mRNA microarray data. Predicted miRNA-target relations from integrated analysis were evaluated according to different criteria: Criteria which can be considered for mRNA-miRNA selection include: the total number of miRNA targets per miRNA, the number of transcription factors per miRNA, enrichment of targets in gene expression clusters, or gene ontology terms of all selected miRNAs or miRNA specific target enrichments, as well as the number of collective miRNA targets. The workflow allowed multi-aspect based interpretation of the results, reduced the quantity of miRNA-mRNA target relations, and increased the prediction quality with respect to potential biological implications.

After 24 h of control myoblast differentiation or concomitant treatment with TNF-α or IGF1 samples were profiled for mRNA and miRNA expression. The expressions of selected genes were validated by qPCR (data not shown). The expected opposite effect of TNF-α and IGF1 treatment on myoblast differentiation was verified at the level of gene expression (S1 Fig), signal transduction pathway association enrichment (S1 Table), as well as miRNA expression and cell morphology [36]. Moreover, we selected 21 miRNAs of interest (S2 Fig) which were detected on two independent miRNA profiling platforms including growth- as well as differentiation-associated miRNAs. Results from the integrative analysis of target prediction, mRNA profiling, and miRNA profiling were evaluated by criteria such as pathway enrichment analyses of targets and the number of targets per miRNA (Fig 1).

### Data-driven integration of miRNA and mRNA expression reduced the number of predicted miRNA-mRNA relations

Integrative analysis reduced the sum of predicted miRNA-mRNA interactions by a factor of more than 20 compared to the sum of targets derived from sole *in silico* prediction (miRanda) (S3A Fig). Moreover, the sum of predicted target interactions per miRNA was reduced by a similar factor (S3B Fig). On average miRanda predictions analysis resulted in about 6000 targets per miRNA compared to integrative analysis of miRNA expression data which resulted on average in about 320 target predictions per miRNA. The integration of both miRNA expression datasets (intersection dataset) reduced the number of predicted target interactions to around 140 targets per miRNA. In the following, we present data derived from the miRNA expression intersection dataset.

### MiRNA targets were involved in cell cycle and axon guidance

**Fig 2:**
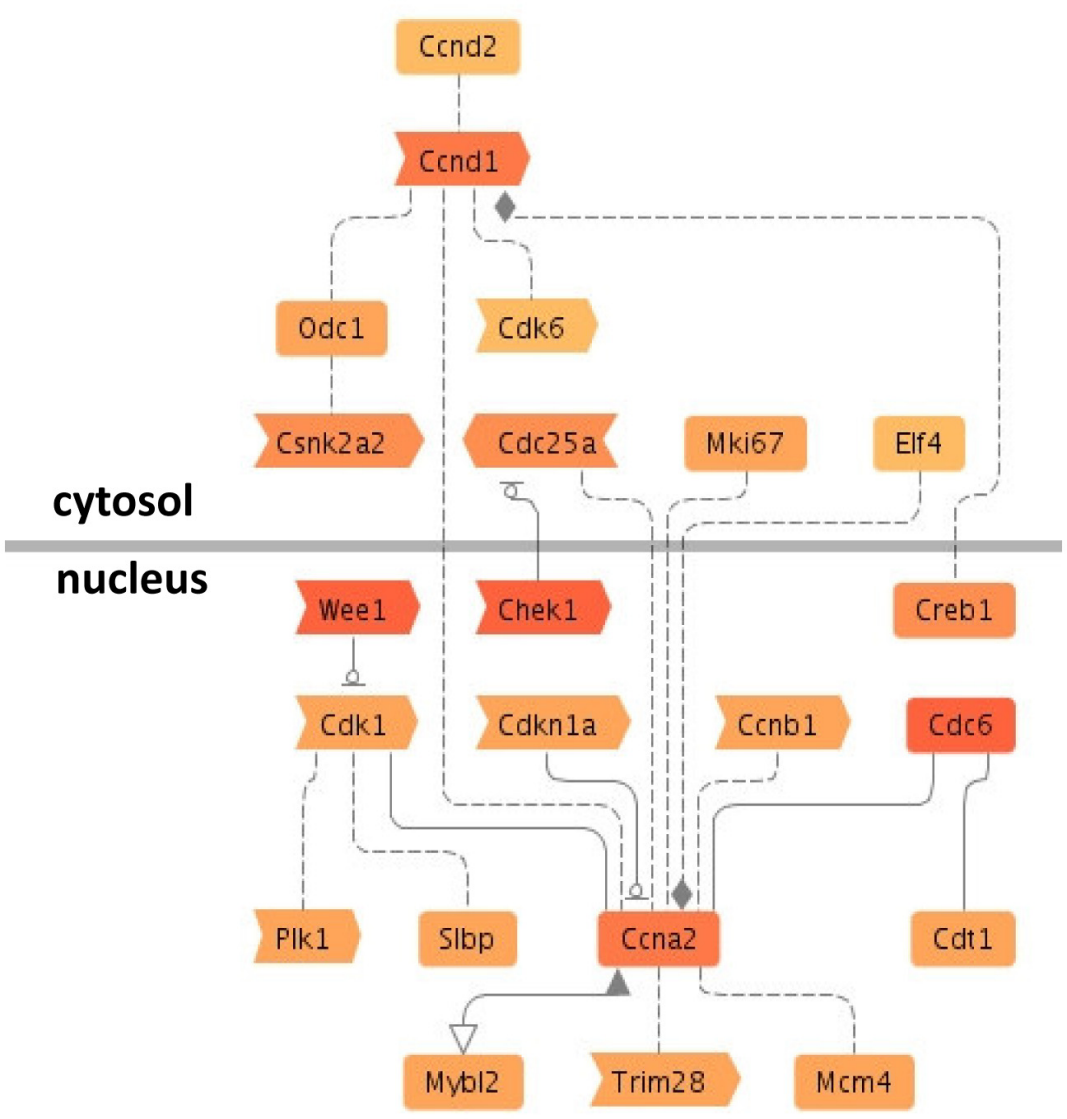
miRNAs target the cyclin A2 pathway. Inversely associated targets had a function in certain enriched signal transduction pathway associations by co-citation on sentence level according to Genomatix Pathway System (GePS) analysis. The results of signal transduction pathway association analysis are exemplarily depicted for cyclin A2 (see S2B Table for a complete list of pathway associations). The red shading intensity increases with the number of miRNAs which target the respective gene.

We analyzed enrichment of pathways or disease terms based on the inversely associated miRNA targets for the set of the selected and inter-platform validated 21 miRNAs. KEGG pathway analyses and signal transduction pathway association analysis showed that targets which were associated with e.g. cell division-related pathways, axon guidance, and the p53 signaling pathway were among the most significantly correlated miRNA target enriched pathways (Table 1 and S2A Table). Enrichment analysis of disease terms based on co-citation showed that the inversely associated miRNA-targets were linked to neoplasm and carcinoma (S2B Table). The interrelations of pathway cyclin A2 associated genes showed that based on co-citation some targets were highly networked such as Ccna2, Cdk1, and Ccnd1 (Fig 2). In addition to that, some genes such as Wee1, Chek1, Cdc6, Ccna2, and Ccnd1 were associated with several enriched pathways (S2A Table) and were predicted to be targeted by several inversely regulated miRNAs including foremost miR-322-5p, miR-206, and miR-503.

**Table 1:** Target enrichment in distinct KEGG pathways.

Term	KEGGID	Pvalue	# Genes (observed)	# Genes (total)
Cell cycle	04110	2.55E-09	45	123
Axon guidance	04360	1.87E-08	45	130
DNA replication	03030	4.93E-07	18	35
Ribosome biogenesis in eukaryotes	03008	1.23E-06	28	74
RNA transport	03013	2.62E-06	46	156
Oocyte meiosis	04114	2.93E-06	36	111
p53 signaling pathway	04115	4.46E-06	25	66
Neurotrophin signaling pathway	04722	9.54E-05	36	128
GnRH signaling pathway	04912	0.000208	29	99
Progesterone-mediated oocyte maturation	04914	0.000252	26	86
Purine metabolism	00230	0.000799	40	162
Glioma	05214	0.000992	20	65
Homologous recombination	03440	0.001101	11	27
ErbB signaling pathway	04012	0.001802	24	87
Gap junction	04540	0.001802	24	87
Prostate cancer	05215	0.002513	24	89
Mismatch repair	03430	0.003019	9	22
VEGF signaling pathway	04370	0.003324	21	76
One carbon pool by folate	00670	0.004161	8	19
Focal adhesion	04510	0.004651	44	199

Negatively associated miRNA targets are significantly retrieved in enriched KEGG pathways calculated based on the amount of observed and the total amount of genes. Only the top 20 terms with p-values < 0.01 are depicted.

### Transcription factors involved in TGF-ß signaling, development-related pathways or cell cycle pathways were targeted

Since modulation of transcription factor abundance is expected to result in significant transcriptomic changes we conducted enrichment analysis of signal transduction pathway associations of targeted transcription factors only (S3 Table). Skeletal muscle differentiation, TNF-α or IGF1 response associated miRNAs regulated transcription factors involved in TGF-β (S4 Fig) and mothers against decapentaplegic homolog (SMAD) signaling as well as development or cell cycle regulation, such as notch or cyclin signaling (S3 Table).

### Gene expression clusters contained miRNA targets of certain pathways

**Fig 3:**
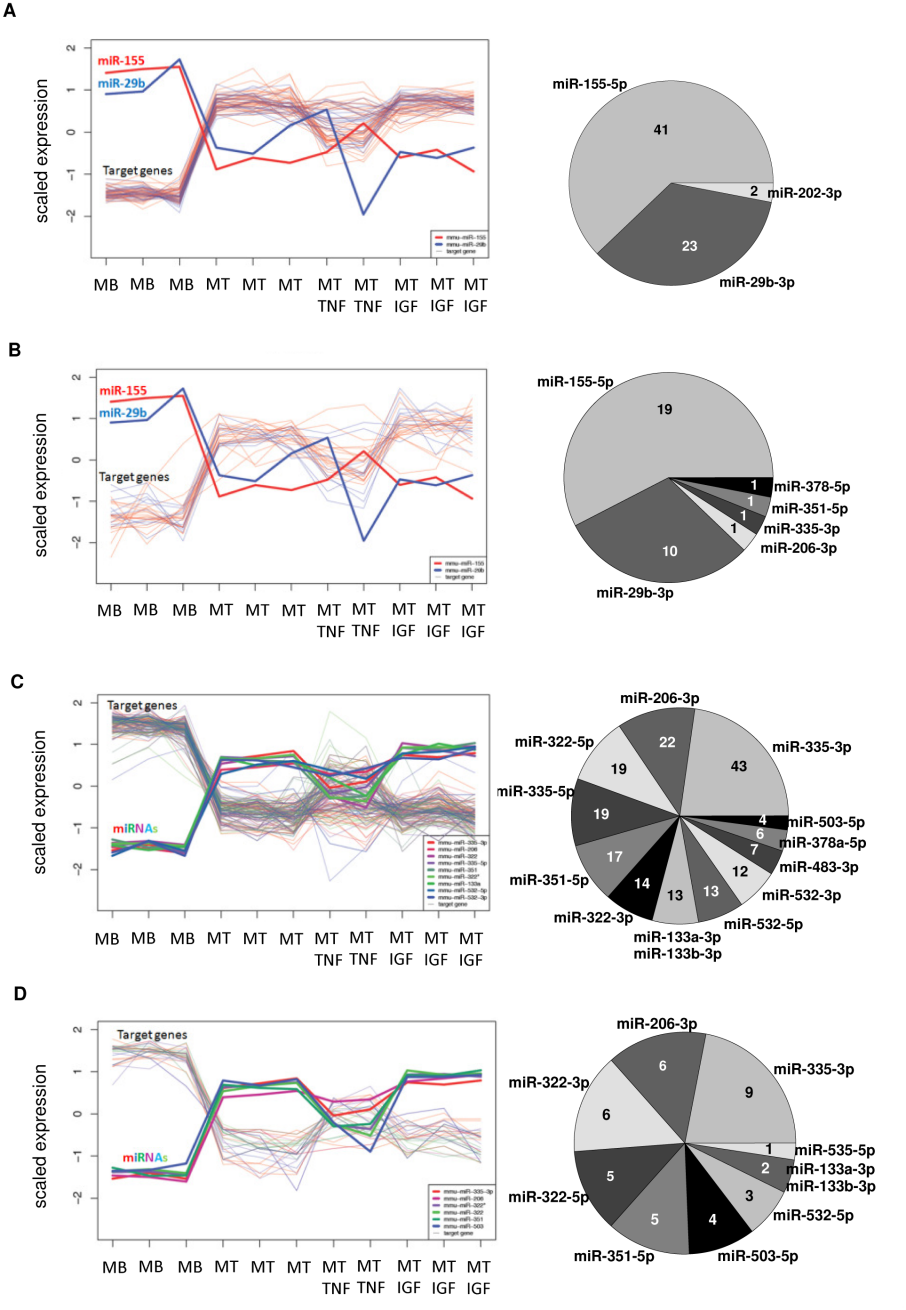
Gene expression clusters are targeted by distinct miRNA groups. Scaled expression (mean subtraction and division by standard deviation) levels of mRNA targets and their respective predicted and inversely associated miRNAs were depicted. Pie charts represent the relative number of mRNA targets per miRNA within the respective gene expression cluster. Absolute target numbers per miRNA within the gene expression cluster were given as well. Self-organizing tree algorithm clusters containing up-regulated genes in **(A)** early (24 h) or **(B)** late (72 h) myogenic differentiation, and genes which were **(C)** down-regulated in early or late myogenic differentiation, or **(D)** down-regulated in later (72 h) myoblast differentiation (72 h data not shown). A list of the outlined miRNA-mRNA relations was deposited in S4 Table.

We tested whether targets of specific miRNAs are over-represented in certain gene expression clusters. Self-organizing tree algorithm (SOTA) analysis revealed that cohorts of genes which were upregulated during early or late myogenic differentiation were mostly targeted by miR-155-5p or miR-29b-3p (Fig 3A and 3B; S4A and S4B Table). MiR-155-5p and miR-29b-3p targeted early differentiation upregulated genes (Fig 3A and S4A Table) which are retrieved in pathway associations such as semaphorin, cannabinoid receptor, and adenylate cyclase signaling (S5A Table) as well as the gene ontology terms steroid biosynthetic process and skeletal muscle tissue development (S5B Table). Moreover, miR-155-5p and miR-29b-3p targets were overrepresented in the cluster containing genes which were upregulated during later myoblast differentiation (Fig 3B and S4B Table). These targets had a function in calcineurin (protein phosphatase 3) and protein kinase A signaling (S5C Table) as well as biological processes such as regulation of ERK1 and ERK2 cascade, MAPK cascade, and regulation of chemokine production (S5D Table). In contrast to clusters containing upregulated promyogenic genes, clusters with down-regulated genes in early or late myogenic differentiation were enriched for miR-335-3p, -206-3p, -322-5p, -335-5p, -351-5p, -322-3p, -133a-3p, -133b-3p, -532-5p and miR-532-3p targets (Fig 3C and S4C Table). Genes targeted by miR-335-3p predominate the cluster of down-regulated genes in early or later differentiation (Fig 3C and S4C Table). MiRNA targets within the cluster of down-regulated genes during differentiation were associated with, for example, SMAD, hypoxia inducible factor 1 (alpha subunit), parathyroid hormone related protein, and TGF-β (S5E Table). Moreover, miRNA targets of this cluster (Fig 3C and S4C Table) were involved in biological processes such as anatomical structure and blood vessel morphogenesis, locomotion, cell differentiation, migration and proliferation (S5F Table). Finally, clustering genes which were down-regulated in later myoblast differentiation were enriched for miR-335-3p, -206-3p, -322-3p, -322-5p, -351-5p, and miR-503-5p targets (Fig 3D and S4D Table) which were associated with, for example, nuclear factor like 2, breast cancer 1 and 2 (early onset), tumor protein p53, cell division cycle 25C (S5G Table). Accordingly, biological processes such as microtubule cytoskeleton organization, DNA metabolic process and regulation of histone H3-K9 acetylation were enriched (S5H Table). In summary, results from joint miRNA-mRNA analysis indicated that cluster of gene expressions contained targets of distinct miRNAs and that these targets had a function within particular pathways and biological processes related to myoblast differentiation.

### Target quantity and versatility of biological functions was miRNA-specific

**Fig 4:**
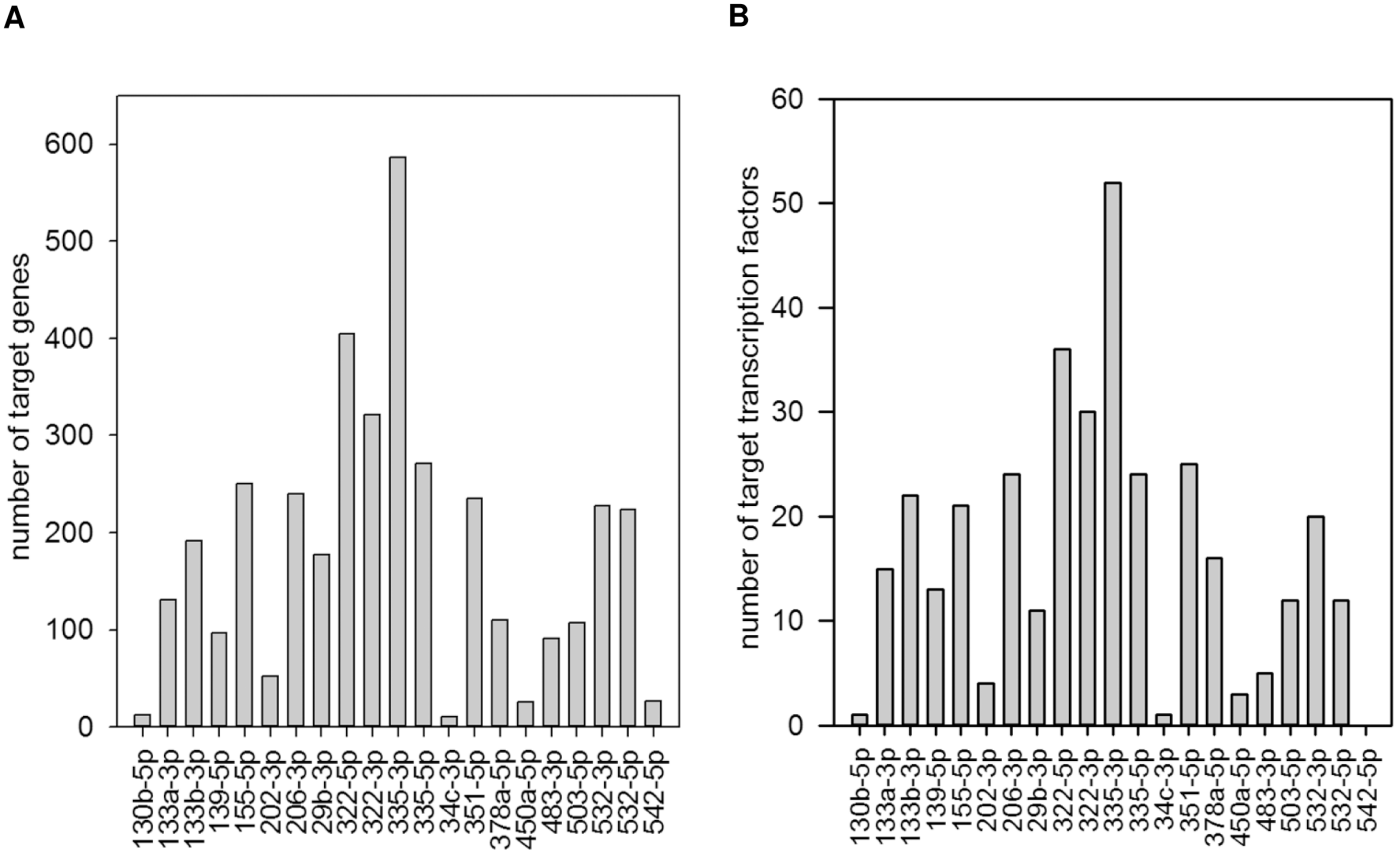
Abundance of targets is miRNA-specific. Joint analysis of miRNA and mRNA expression data and target prediction revealed miRNA-specific numbers of inversely associated **(A)** targets and **(B)** transcription factors.

The number of inversely associated targets during myoblast differentiation and response to TNF-α or IGF1 was miRNA-specific. MiR-335-3p, miR-322-5p, and miR-322-3p had the highest number of targeted and inversely associated genes and transcription factors (Fig 4A and 4B). The versatility of biological functions was miRNA-specific. MiR-206-3p targets had a function in enriched pathways known to be of significance for myogenic differentiation such as e.g. TGF-β, cyclin A2, caveolin1, and focal adhesion kinase (S6A Table). MiR-322-3p targets had a function, for example, in cancer-related pathways, cell division cycle, ataxia telangiectasia and Rad3 related, and tumor protein p53 (Tp53) (S6B Table). Moreover, miR-322-5p showed a remarkable overrepresentation of targets involved in cell division-associated pathways such as cyclins A2, B1, and D1 as well as cyclin dependent kinase, as well as cell division cycle 2 and 25c (S6C Table). MiR-335-3p targets were associated with e.g. cell division cycle 2, fibroblast growth factor, and TGF-β signal transduction (S6D Table). Furthermore, miR-335-5p target enrichment analysis revealed cyclin dependent kinase inhibitor 1 and cyclin A2 associated genes (S6E Table). In contrast, miR-351 targets were functional in the extracellular matrix such as matrix metalloproteinase Mmp12 or Adam17 (S6F Table). MiR-503-5p revealed targets such as cyclins, ataxia telangiectasia and Rad3 related (Atr), cell division cycle, and cancer-related genes, as well as Tp53 (S6G Table). Thus, miR-322-3p and miR-503-5p targeted a similar spectrum of pathways in skeletal muscle differentiation. Moreover, miR-133a-3p and miR-133b-3p targets were enriched for V Akt murine thymoma viral oncogene homolog 1 (Act1) and small GTP binding protein Rac associated genes (S6H Table) while miR-155-5p targets were part of calcineurin pathway associations (S6I Table). Taken together, some miRNAs targeted genes which had a function in rather distinct pathways while other miRNAs targeted genes which were functionally related, such as cell cycle regulation.

### Pathways and GO terms were targeted by several miRNAs with distinct preference

The number of miRNA-mRNA interactions per miRNA was summarized for selected enriched KEGG pathways or GO terms. Our data revealed that the number of targets per miRNA within an enriched pathway or GO-term greatly varied indicating specific as well as common functions of individual miRNAs (S5 Fig). All of the twelve selected miRNAs had at least more than 10 targets involved in cell cycle regulation but miR-322-5p and miR-322-3p appeared to be the dominating miRNAs involved in regulation of this pathway. Furthermore, kinase activity was predominantly targeted by miR-322-5p. Moreover, regulation of cell motility was mainly targeted by miR-335-3p and miR-206-3p (S5 Fig).

### A subset of myoblast-differentiation associated miRNAs might participate in collective post-transcriptional regulation of gene expression

**Fig 5:**
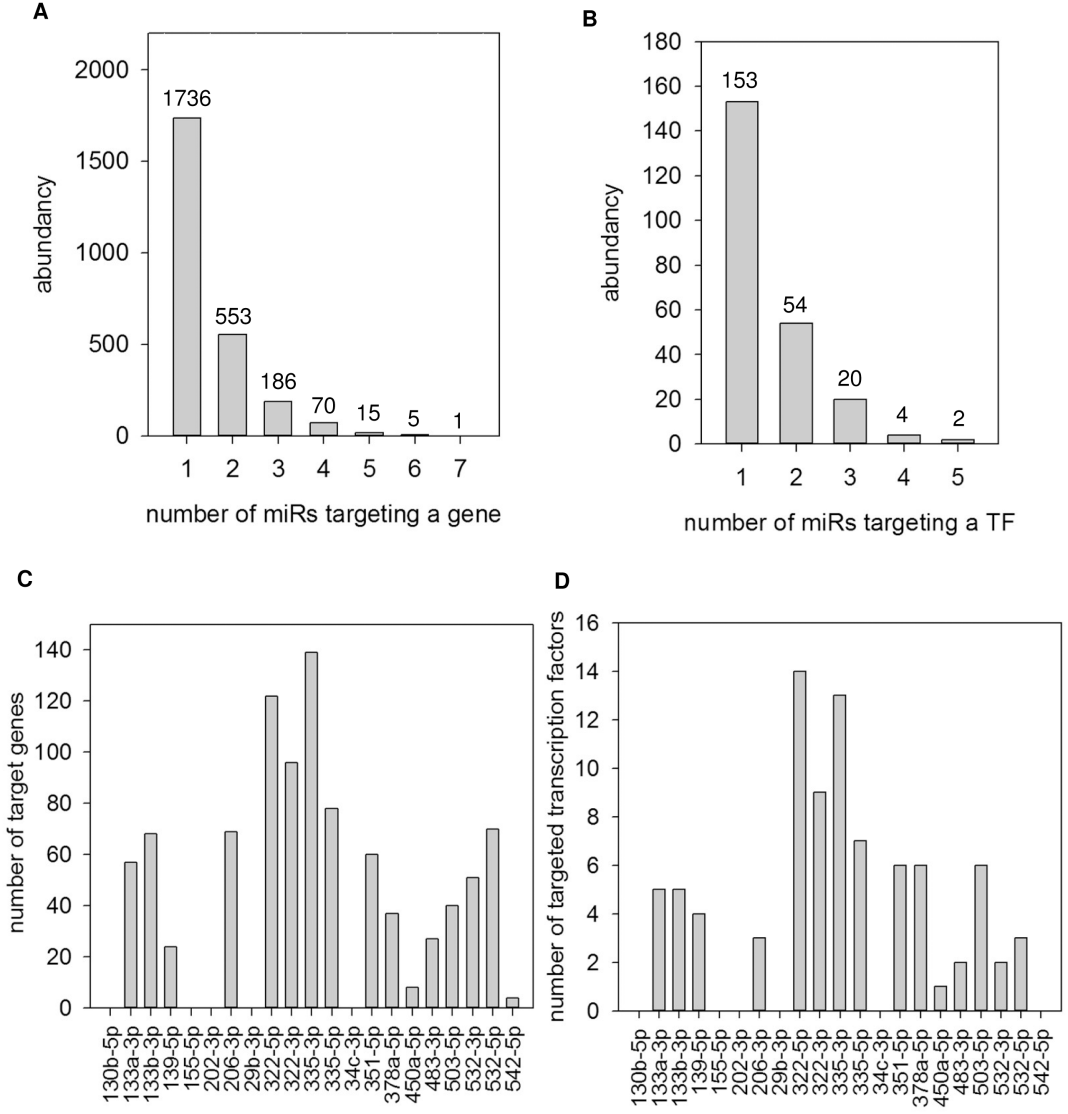
Collective target regulation by miRNAs. The abundance of **(A)** genes or **(B)** transcription factors which were targeted by a specific number of miRNAs of the observed miRNA set is shown. **(C)** The number of genes targeted by at least two other miRNAs (resulting in at least three miRNAs per gene) of the 21 miRNA-subset per individual miRNA and **(D)** the number of transcription factors targeted by at least two other miRNAs of the 21 miRNA-subset per individual miRNA are depicted. The abundance of miRNA-target interactions indicates collective target regulation by a subset of muscle expressed miRNAs.

**Fig 6:**
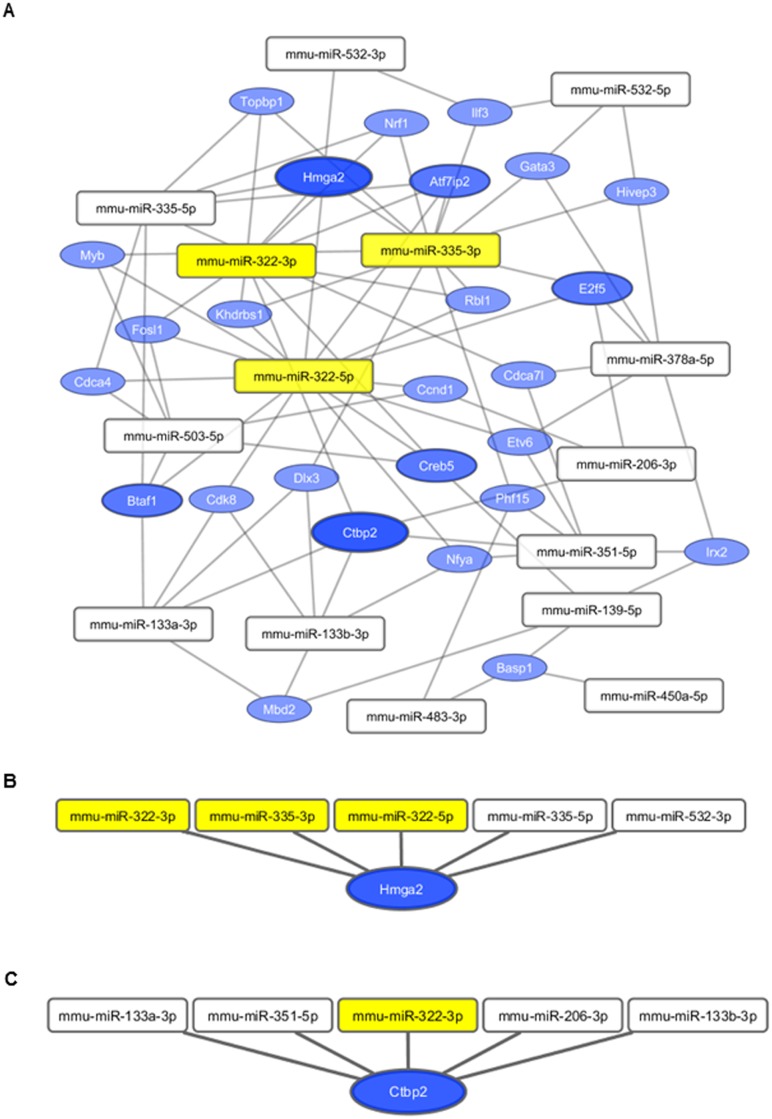
Cooperative targeting of transcription factors. **(A)** MiRNA-target network of transcription factors which are targeted by at least three miRNAs. Edges connect the respective miRNA with its predicted and inversely associated transcription factors which were represented by nodes. The bigger the size of the node the more edges did the target have indicating collective targeting by several miRNAs. MiRNAs highlighted by yellow had the highest number of collectively targeted transcription factors. **(B)** Hmga2 and its targeting miRNAs, **(C)** Ctbp2 with targeting miRNAs.

The abundance of targets which were inversely associated with several miRNAs suggested collective target regulation by a subset of muscle expressed miRNAs (Fig 5A, 5B, 5C and 5D). More than 200 genes (Fig 5A) and more than 20 transcription factors (Fig 5B) were predicted to be targeted by at least three miRNAs. MiRNAs which mainly participated in collective gene targeting were miR-335-3p, miR-322-5p, and miR-322-3p (Fig 5C). As transcription factors are powerful master regulators, we analyzed which miRNAs particularly were involved in collective regulation of transcription factors. MiR-322-5p, miR-335-3p, and miR-322-3p were primarily involved in the concerted regulation of transcription factors (Figs 5D and 6A). Two transcription factors, Hmga2 and Ctbp2, were cooperatively targeted by five miRNAs (Fig 6B and 6C). Genes which were targeted by at least three different miRNAs were retrieved in signal transduction pathway associations such as cyclin dependent kinase inhibitor, cell division cycle, thrombospondin, cyclin signaling, or ataxia telangiectasia (Table 2). Genes which were collectively targeted by miRNAs were assigned to GO terms such as metabolic process, mRNA splice site selection, positive regulation of cell migration, and positive regulation of cell motility (S7A Table). Amongst others, the disease terms tendinopathy and neoplasms were enriched (S7B Table).

**Table 2:** Collective targeting of cell cycle genes.

Pathway	P-value	List of observed genes
CYCLIN DEPENDENT KINASE INHIBITOR 1	2.65E-04	Kras, Bcl2, Chek1, Cdc6, Wee1
CELL DIVISION CYCLE 2, G1 TO S AND G2 TO M	5.94E-04	Bcl2, Chek1, Hmmr, Cdc6, Wee1
THROMBOSPONDIN 1	9.09E-04	Kras, Ptk2, Vegfa
CYCLIN B1	1.97E-03	Bcl2, Chek1, Wee1
CYCLIN A2	2.88E-03	Chek1, Cdc6, Wee1
ATAXIA TELANGIECTASIA AND RAD3 RELATED	4.65E-03	Chek1, Cdc6, Mcph1
WEE1 HOMOLOG	6.02E-03	Chek1, Wee1
BMX NON RECEPTOR TYROSINE KINASE	6.63E-03	Ptk2, Vegfa
CYCLIN DEPENDENT KINASE	7.20E-03	Kras, Chek1, Smurf1, Cdc6, Wee1

Genes which were inversely associated and potentially targeted by at least three different miRNAs are retrieved in signal transduction pathway association (GePS analysis) such as cell cycle regulation and are depicted with corresponding p-values.

### Integrative analysis of miRNA and mRNA data and its evaluation increased the quality of miRNA target predictions

The integrative analysis of holistic miRNA and mRNA expression data improved the quality of miRNA target prediction. About 1% of the targets identified by joint mRNA and miRNA data analysis had been validated based on bioinformatics database entries in miRTarBase (http://mirtarbase.mbc.nctu.edu.tw/), miR2Disease (http://www.mir2disease.org/), miRecords (http://mirecords.biolead.org/). When applying evaluation criteria such as the number of targets or transcription factors per miRNA, GO term or pathway enrichment of targets, or degree of collective target regulation by several miRNAs (summarized in Fig 1) the selection of miRNA-target relations could be improved to containing about 14% validated targets (S6 Fig). The improved recovery rate of yet validated targets might indicate an amendment of selecting biological significant miRNA-mRNA relations by applying simultaneous miRNA-mRNA data analysis in combination with investigator based evaluation criteria in a specific physiological or experimental context.

To further evaluate the quality of the computationally verified miRNA-target relations, we compared our network to experimentally determined miRNA-target relations from Starbase (http://starbase.sysu.edu.cn/), which holds the results from various HITS-CLIP and PAR-CLIP experiments. We downloaded all miRNA-target relations, which were also predicted by both, TargetScan and miRanda. We then aimed to assess whether this set of experimentally determined miRNA-target relations is overrepresented among our generated intersection network in comparison to all predicted relations, which were subject to our expression-based filtering approach. We observed that 12.92% of the relations in our generated intersection network were experimentally validated using HITS-CLIP or PAR-CLIP, whereas this was the case only for 10.67% of all predicted relations. Application of a one-sided Fisher’s exact test yielded a p-value of 0.023 indicating a statistically significant overrepresentation.

## Discussion

Targets of regulated miRNAs might enhance or ameliorate the effect of TNF-α or IGF1 treatment on the differentiation capacity of skeletal myoblasts. In light of the high false positive rate of *in silico* miRNA target prediction [13] it is advantageous to employ and integrate information from the miRNA and mRNA profiling data together with bioinformatics prediction tools. However, existing studies about the impact of inflammatory or anabolic stimuli on skeletal myoblast differentiation focus on either mRNA or miRNA expression levels. This study expanded the methodology to view the results in the context of joint mRNA and miRNA analysis in myoblast differentiation under cytokine or anabolic influence and facilitated the elucidation of the post-transcriptional regulatory networks and the prioritization of potential miRNA-mRNA interaction pairs. To date, there has been only one other study using integrative mRNA-miRNA analysis in the context of myoblast differentiation [37].

By applying integrated mRNA and miRNA analysis together with target prediction we reduced the complexity of predicted miRNA-mRNA relations on average by more than 18-fold compared to pure in silico prediction. When focusing on the miRNA subset which overlapped on both miRNA profiling platforms we reduced the complexity by more than 40-fold. Thereby, we enlarged the list of highly potential targets of miRNAs implicated in skeletal myoblast differentiation foremost miR-155, miR-206, miR-322-3p/-5p, miR-335-3p/-5p, miR-351, and miR-532-3p/-5p. Thus, the role of e.g. miR-155 in myogenic target regulation has been underestimated so far. Moreover, this is the first study identifying a predominant functional role of miR-335-3p in skeletal myoblast differentiation on the basis of simultaneous analysis of miRNA and mRNA expression data.

Furthermore, we evaluated the predicted targets based on criteria such as the number of targets or transcription factors per miRNA, the enrichment of targets in pathways and gene expression clusters, and the indication of cooperative target regulation by several distinct miRNAs. We corroborated an anti-myogenic role of miR-155-5p in skeletal muscle cell differentiation. So far, it had been known that miR-155 overexpression repressed expression of MEF2A [38], which is an important pro-myogenic transcription factor [39,40]. Moreover, miR-155 overexpression down-regulated WEE1 (WEE1 homolog-*S*. *pombe*), a kinase that blocks cell-cycle progression [38]. However, exit from the cell cycle is a prerequisite for terminal myoblast differentiation [41].

In addition, our data corroborated a function of miR-29b-3p in muscle differentiation. It has been reported that miR-29b-3p was down-regulated in Myotonic Dystrophy Type-1 biopsies compared to controls [42]. Our data support the hypothesis that down-regulation of miR-29b-3p promotes myoblast differentiation. However, expression studies in mice with chronic kidney disease showed that an increase in miR-29 improved the differentiation of myoblasts into myotubes [43], which appears to contradict our findings.

On the other hand, our study provided new insights into the biological implications of miR-155-5p and miR-29b-3p in skeletal muscle cell differentiation. Furthermore, we showed that these two miRNAs were not primarily involved in cooperative target regulation. Instead, a decrease in miR-155-5p expression was associated with an increased calcineurin (protein phosphatase 3) related gene expression. Latter is in good agreement with the known up-regulation and pro-differentiation function of calcineurin in myoblast differentiation [44–46] as well as muscle regeneration [47] by activating MEF2 and MyoD and inducing myogenin [48,49].

In addition, we corroborated the expression of myoblast differentiation promoting miRNAs, such as miR-322-3p, miR-322-5p, miR-335-3p, and miR-335-5p, which were among the miRNAs showing the highest number of inversely associated targets and transcription factors as well as the highest degree of cooperative target regulation. In contrast to anti-myogenic miR-155-5p and miR-29b-3p, the pro-myogenic miR-322-3p, miR-322-5p, miR-335-3p, and miR-335-5p were involved in down-regulation of, for example, cell cycle or growth related pathways. There has been little evidence in the literature about the biological implications of miR-335-3p and miR-335-5p in myoblast differentiation or its response to TNF-α or IGF1, respectively. MiR-335-3p was differentially expressed in myogenic progenitor cell differentiation [50]. However, the biological implications remained unknown. Our study suggests that miR-335-3p was involved in target regulation of cell division related genes as well as fibroblast growth factor or TGF-β signaling. Thus, we provide new evidence for a significant role of miR-335-3p in myoblast differentiation. Similarly, our data revealed involvement of miR-335-5p in cyclin dependent kinase inhibitor and cyclin A2 regulation. MiR-335-5p was likely functionally relevant as the cyclin A2 pathway was the top enriched pathway when analyzing all investigated inversely associated miRNA targets. Furthermore, it had been shown that miR-335-5p was up-regulated following myoblast differentiation [51] and that miR-335-5p was induced in mdx mice and DMD patients as well as newly formed myofibers during postischemic regeneration [51] and primary muscle disorders [52,12]. Interestingly, our data revealed enrichment of TGF-β associated targets for miR-335-3p. However, other studies indicated that miR-335-5p targeted genes in the TGF-β non-canonical pathways in neuroblastoma cells [53]. Furthermore, miR-335-5p had been reported as tumor suppressor [54,55] or tumor promoter [56]. Besides, miR-335-5p was a pro-apoptotic and antimitogenic factor [57] in the brain and induced cell cycle arrest in human cancer cells [58] or suppressed cell proliferation in prostate cancer [59]. Thus, we conclude, based on our target enrichment analysis and known functions within other cell types, that miR-335-5p might play a role in cell cycle withdrawal during myoblast cell differentiation. In addition, we revealed that miR-335-5p had the highest number of potential targets and the highest number of targeted transcription factors. We hypothesize that miR-335-3p and miR-335-5p played a significant role in post-transcriptional regulation of gene expression in differentiating myoblasts and TNF-α response.

Furthermore, our data showed that inversely associated miR-322-3p targets were involved in the regulation of, for example, cell cycle, cancer or ataxia telangiectasia. These findings provide new indications into the biological function of miR-322-3p as there have been no explicit functional studies on miR-322-3p in muscle cells yet. In contrary, it has been reported that miR-322-5p and miR-503 were induced during myogenesis and promoted cdk2 inhibition by down-regulating Cdc25A, the phosphatase responsible for removing inhibitory phosphorylation of cdk2 [60]. We corroborated inverse association of Cdc25A and miR-322-5p. We showed that Cdc25A was associated with cyclin signaling, cell division cycle, and cyclin dependent kinase pathways which is in harmony with studies in other cell types describing a role of miR-322-5p in the regulation of the cell cycle and cell growth [61–63]. Moreover, we confirmed targets of miR-322-5p in myoblast differentiation which have been published in other tissues and cell types including Chk1 [61], Wee1 [62], cyclin D1 [64], and cyclin E1 [65]. However, inverse association of miR-322-5p and protein level but not mRNA abundance of cyclin E1 was observed [65]. We showed inverse association of cyclin E1 mRNA. In addition, our data revealed a negative impact of TNF-α exposure on the expression of pro-differentiative miR-322-3p. Latter could be explained by studies showing that miR-322-5p expression was inhibited by NF kappa B activity [66]. However, in other inflammatory contexts, such as TWEAK treatment of myotubes, miR-322-5p was upregulated [67]. Consistent with our findings, miR-322-5p was upregulated in anti-inflammatory drug treated myotubes in a model of dexamethasone induced muscle atrophy [68]. Thus, our data affirmed that miR-322-5p was sensitive to pro-inflammatory stimuli in the context of skeletal muscle.

In summary, we presented several new inversely associated genes of for example miR-335-3p, miR-335-5p, miR-322-3p, and miR-322-5p with emphasis on the regulation of the cell cycle-related pathways. Furthermore, we showed that inter alia miR-335-3p, miR-335-5p, miR-332-3p, and miR-332-5p were downregulated by TNF-α treatment whereas IGF1 had no significant impact on expression levels of these miRNAs. A study by Dmitriev et al. [37], which used integrative analysis of mRNA and miRNA expression data during human myoblast differentiation, did not identify differential regulation of miR-335 or miR-322 (miR-424 in human). Moreover, Dmitriev et al. [37] identified different functional classes of targets compared to our study. However, miRNA targets associated with e.g. cell cycle regulations were retrieved in enriched pathways as well. Differences between our study and the results published by Dmitriev et al. [37] may result from species differences.

One of the challenges to understand miRNA-mediated regulation is to identify co-regulating miRNAs that simultaneously regulate their target genes in a network perspective [69]. MiRNAs with similar characteristics such as co-expression [17] or concordance between targets [69] are predicted to target a higher number of mRNAs cooperatively than unrelated miRNAs [17]. This study revealed that several targets were inversely associated to at least three potentially targeting miRNAs, which might indicate that these miRNAs synergistically acted as modulators of myoblast cell differentiation and response to TNF-α. Particularly, miR-335-3p, miR-322-5p, and miR-322-3p predominated collective regulation of genes including transcription factors. Based on enrichment analyses these miRNAs seemed to function cooperatively in, for example, cyclin dependent kinase inhibitor and cyclin signalling, as well as cell division cycle regulation. In concordance with these functional indicators we found Hmga2 which was cooperatively targeted by five inversely associated miRNAs. Hmga2 was highly and specifically expressed in proliferating skeletal myoblasts and directly regulated the RNA-binding protein IGF2BP2 during myoblast differentiation [70]. This study highlighted the massive and redundant targeting of Hmga2 expression by miRNAs during myotube formation. It had been confirmed that Hmga2 declined during fusion of myoblasts into myotubes and that Hmga2 overexpression promoted myoblast growth and that the HMGA2-IGF2BP2 axis functioned as a key regulator of skeletal muscle development [70]. Another transcription factor, Ctbp2, was targeted by five miRNAs which strongly suggested significant biological implications in myoblast differentiation. Moreover, CtBP proteins functioned as corepressors, which repressed transcription by interacting with ZEB [71], a negative regulator of muscle differentiation [72]. Interestingly, it had been verified that the CtBP/ZEB complex was efficiently regulated by the miR-141-200c cluster which simultaneously targeted several protein components of the protein complex [16]. The study of Sass et al. [16] indicated a coordinate posttranscriptional regulation of protein complexes by miRNAs. Our data support the orchestrated regulation of an individual component of the complex, namely Ctbp2, by different miRNA families. Taken together, our study highlighted the combinatorial effects of myoblast differentiation-associated miRNAs outlining a complex post-transcriptional regulatory network. Moreover, cooperativity of miRNAs was indicated by co-expression, their shared transcription factors, as well as partly functional coherence of target genes. However, it remains an open question whether the miRNAs function rather synergistically or additive in post-transcriptional target regulation of skeletal muscle cell differentiation and its response to TNF-α or IGF1. Nevertheless, it is expected that all inversely associated miRNAs which may collectively target mRNAs have a high probability to be required for fine-tuning gene expression [16].

Besides the valuable benefits of the suggested integrative approach the method bears some limitations with respect to the validity of mRNA-miRNA relationship predictions. Integrative analysis of mRNA and miRNA profiling data cannot entirely exclude false positives as some gene regulations might be due to indirect effects other than miRNA regulation such as transcription factor regulation. Therefore, cross-linking immunoprecipitation data derived from the same set of experiments are needed to holistically validate the miRNA-target interactions predicted by our method. However, we were able to show that our integrative analysis of expression data can yield a more reliable set of miRNA-target relations in terms of experimental validation than sequence-based *in silico* target predictions only.

## Conclusions

To our knowledge, this is the largest transcriptomic analysis of the impact of TNF-α and IGF1 on *in vitro* skeletal myoblast differentiation. Moreover, we derived indications for functional mRNA-miRNA relationships by integrated data analysis and narrowed down the complexity of predicted miRNA-mRNA relations. We identified significant involvement of miRNAs which have not been described as major players in post-transcriptional regulation of myogenic differentiation yet. Moreover, our data suggest that miRNAs exert joint regulatory functions on gene expressions. The consideration of the miRNA-specific level of cooperative target regulation may facilitate the selection of promising miRNA candidates for therapeutic interventions.

## Supporting Information

S1 Fig Principal component analysis revealed treatment effects. Principal component analysis of gene expression analysis during skeletal myocyte differentiation with control, TNF-α, and IGF1 treatment. Myoblasts were shown in light blue. Myotubes were depicted in green. Myotubes with TNF-α treatment were shown in red while myotubes treated with IGF1 were presented in dark blue. PC 1 explained most of the variance of myocyte differentiation. PC 2 explained most of the variance induced by TNF-α. PC 3 explained most of the variance caused by IGF1 treatment.
(TIF)

S2 Fig miRNA correlation of selected miRNAs. The miRNA heatmap and cluster analysis of 21 selected miRNAs includes all samples: control, TNF-α and IGF1. Perfect correlation is indicated by a value of 1. The majority of the selected miRNAs shows high correlation with the other selected miRNAs. Two inversely regulated cohorts of miRNAs appear to be involved in differentiation and TNF-α or IGF1 response of murine skeletal muscle cells. Of the selected candidates the majority of miRNAs was jointly upregulated during differentiation.
(TIF)

S3 Fig Reduction of complexity of possible miRNA-mRNA relationships. **(A)** The sum of predicted miRNA-mRNA interactions of a subset of 21 selected differentially expressed miRNAs by computational prediction (miRanda) only or by integrative analysis, respectively. The number of possible miRNA-mRNA interactions was significantly reduced by integrative analysis compared to solely computational prediction. **(B)** Each miRNA showed a specific number of targets predicted by miRanda (black) or as a result of integrative analysis based on miRNA microarray profiling data (red), miRNA qPCR (green) profiling data or the intersection (yellow) of microarray and qPCR data. The average numbers of targeted genes per miRNA were depicted for each prediction method.
(TIF)

S4 Fig Targeted transcription factors in the TGF-beta pathway. Targeted transcription factors with a function in the TGF-beta pathway were depicted with interconnections based on co-citation. Circled transcription factors are significantly regulated during differentiation or TNF-α or IGF1 response.
(TIF)

S5 Fig Enriched pathways and GO terms contained targets of distinct miRNAs. Selected enriched GO terms or KEGG pathways are shown, which were identified based on the analysis of all inversely correlated miRNA-mRNA relations of the 21 miRNA subset. Analyses revealed that the number of targets, which were associated with enriched pathways or GO terms, greatly varied per miRNA indicating specific as well as common functions of individual miRNAs.
(TIF)

S6 Fig Probability of selecting functionally relevant miRNA-target relations. Evaluation of results from integrative analysis increases the probability of selecting miRNA-target relations with a high chance of being functionally relevant. The pie chart illustrates that about 14% of the selected miRNA-target interactions have previously been validated. We selected nine miRNAs and 21 target genes of particular interest resulting in 36 miRNA-mRNA relations based on integrated data analysis. Of these 36 relations five miRNA-mRNA interactions have been validated previously.
(TIF)

S1 Table Signal transduction pathway associations of genes identified by principal components. Table showing enrichment of signal transduction pathway associations of PC 2 and PC 3 with p-values and the list of observed genes.
(XLSX)

S2 Table miRNA target expressions had a function in distinct pathways. Target enrichment in **(A)** genes retrieved in signal transduction pathway associations by co-citation on the sentence level, as well as **(B)** MeSH disease terms (# genes (observed): number of genes of the input set which have the respective annotation; # genes (expected): number of genes expected with the respective annotation which is calculated based on the total number of genes with the respective annotation and the number of genes of the input set with the respective annotation; # genes (total): total number of genes with the respective annotation). Only the top 20 terms with p-values < 0.01 within the respective list were shown.
(DOCX)

S3 Table Pathway enrichment of targeted transcription factors. Enrichment analysis of targeted transcription factors for signal transduction pathway associations by co-citation.
(DOCX)

S4 Table Gene expression clusters and their targeting miRNA-relations. Differentiation- / TNFα- / IGF1-associated miRNAs potentially targeted inversely correlated genes which clustered by self-organizing tree algorithm (SOTA) analysis. Cluster analysis was performed for 0–72 h differentiation / treatment, however, only expression values for 24 h differentiation / treatment were depicted. MiRNA-mRNA relations were depicted for: **(A)** cluster of genes which were up-regulated in very early differentiation, **(B)** cluster of genes which were up-regulated in later differentiation, **(C)** genes which were down regulated during very early or later differentiation, **(D)** genes which were down-regulated later during differentiation.
(DOCX)

S5 Table Enrichment of clusters of miRNA target expressions in gene ontology terms and pathways. SOTA analysis of gene expression data of skeletal myoblast differentiation and TNF-α and IGF1 treatment (0–72 h) revealed clusters of gene sets which were targeted by differentiation-associated miRNAs. Scaled expressions of miRNA-mRNA relations based on integrative analysis were depicted for 24 h differentiation / treatment. Clustered targets were retrieved in signal transduction pathway associations or in GO term ‘biological processes’: **(A)**, **(B)** cluster of genes which were up-regulated in very early differentiation, **(C)**, **(D)** cluster of genes which were up-regulated in later differentiation, **(E)**, **(F)** genes which were down regulated during very early or later differentiation, **(G)**, **(H)** genes which were down-regulated later during differentiation.
(DOCX)

S6 Table Pathway enrichment of targets of selected miRNAs. Enrichment analysis of signal transduction pathway associations of **(A)** miR-206-3p, **(B)** miR-322-3p, **(C)** miR-322-5p, **(D)** miR-335-3p, **(E)** miR-335-5p, **(F)** miR-351-5p, **(G)** miR-503-5p, **(H)** miR-133a-3p/miR-133b-3p, **(I)** miR-155-5p.
(DOCX)

S7 Table Enrichment of collectively targeted genes in GO and disease terms. We showed results for genes targeted by at least three miRNAs and refer to these as collectively targeted genes. Collectively targeted genes were associated with **(A)** GO terms ‘biological processes’. **(B)** The top 20 enriched MeSH disease terms of collectively targeted genes.
(DOCX)

S8 Table MiRNA-mRNA relations of high interest. Table of miRNAs showing the selected miRNA-mRNA relations of high interest based on the selection workflow depicted in Fig 1. Column “predicted to be targeted by….” is based on data of this study whereas column “previous evidence….” is based on literature search.
(DOCX)
