# Supplementary figures and images for: Integrative Analysis of MicroRNA and mRNA Data Reveals an Orchestrated Function of MicroRNAs in Skeletal Myocyte Differentiation in Response to TNF-α or IGF1
Source: PLoS One. 2015 Aug 13;10(8):e0135284. doi: 10.1371/journal.pone.0135284 (PMC4536022; doi:10.1371/journal.pone.0135284)

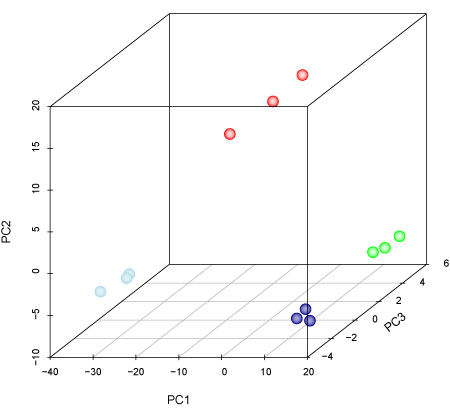

Supplement: S1 Fig — Principal component analysis of gene expression analysis during skeletal myocyte differentiation with control, TNF-α, and IGF1 treatment. Myoblasts were shown in light blue. Myotubes were depicted in green. Myotubes with TNF-α treatment were shown in red while myotubes treated with IGF1 were presented in dark blue. PC 1 explained most of the variance of myocyte differentiation. PC 2 explained most of the variance induced by TNF-α. PC 3 explained most of the variance caused by IGF1 treatment. (TIF) [file pone.0135284.s001.tif]

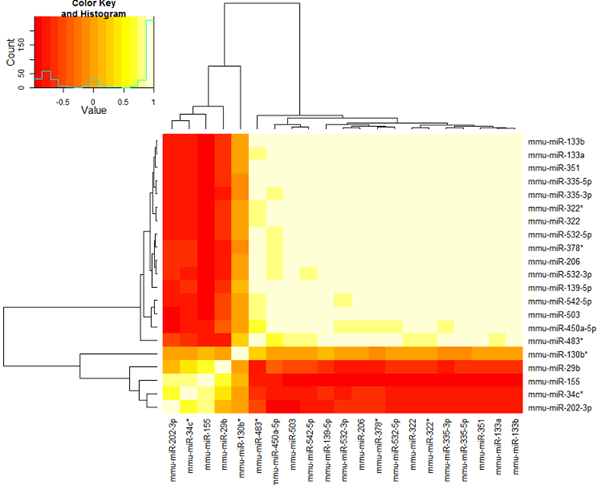

Supplement: S2 Fig — The miRNA heatmap and cluster analysis of 21 selected miRNAs includes all samples: control, TNF-α and IGF1. Perfect correlation is indicated by a value of 1. The majority of the selected miRNAs shows high correlation with the other selected miRNAs. Two inversely regulated cohorts of miRNAs appear to be involved in differentiation and TNF-α or IGF1 response of murine skeletal muscle cells. Of the selected candidates the majority of miRNAs was jointly upregulated during differentiation. (TIF) [file pone.0135284.s002.tif]

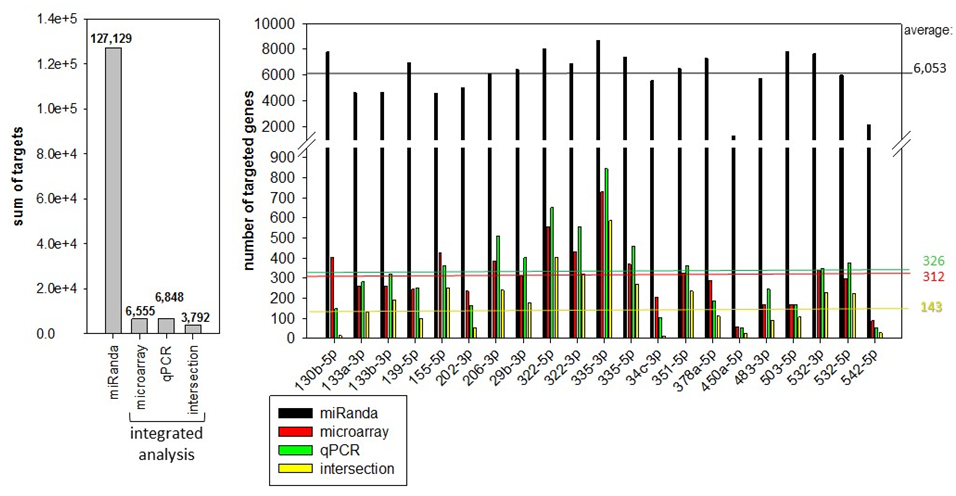

Supplement: S3 Fig — (A) The sum of predicted miRNA-mRNA interactions of a subset of 21 selected differentially expressed miRNAs by computational prediction (miRanda) only or by integrative analysis, respectively. The number of possible miRNA-mRNA interactions was significantly reduced by integrative analysis compared to solely computational prediction. (B) Each miRNA showed a specific number of targets predicted by miRanda (black) or as a result of integrative analysis based on miRNA microarray profiling data (red), miRNA qPCR (green) profiling data or the intersection (yellow) of microarray and qPCR data. The average numbers of targeted genes per miRNA were depicted for each prediction method. (TIF) [file pone.0135284.s003.tif]

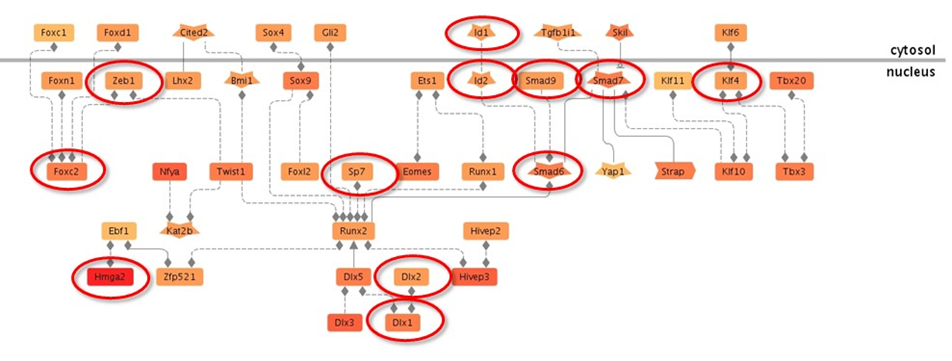

Supplement: S4 Fig — Targeted transcription factors with a function in the TGF-beta pathway were depicted with interconnections based on co-citation. Circled transcription factors are significantly regulated during differentiation or TNF-α or IGF1 response. (TIF) [file pone.0135284.s004.tif]

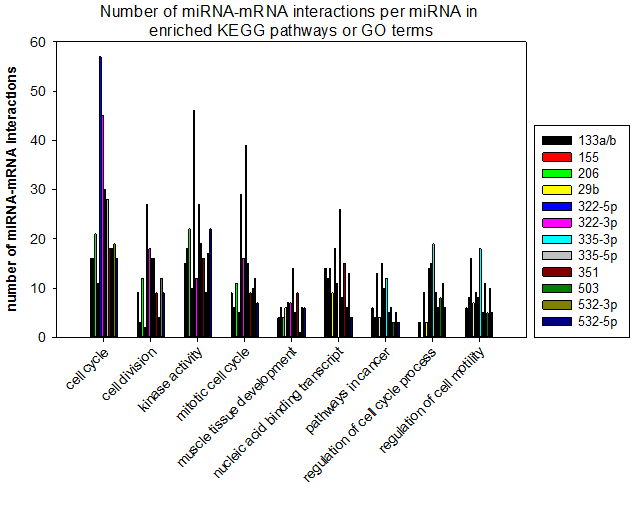

Supplement: S5 Fig — Selected enriched GO terms or KEGG pathways are shown, which were identified based on the analysis of all inversely correlated miRNA-mRNA relations of the 21 miRNA subset. Analyses revealed that the number of targets, which were associated with enriched pathways or GO terms, greatly varied per miRNA indicating specific as well as common functions of individual miRNAs. (TIF) [file pone.0135284.s005.tif]

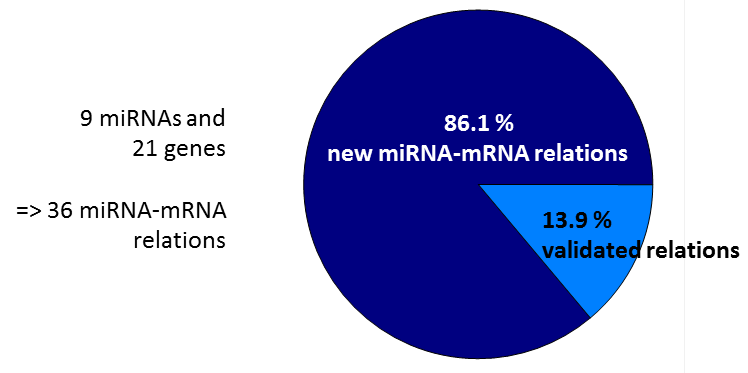

Supplement: S6 Fig — Evaluation of results from integrative analysis increases the probability of selecting miRNA-target relations with a high chance of being functionally relevant. The pie chart illustrates that about 14% of the selected miRNA-target interactions have previously been validated. We selected nine miRNAs and 21 target genes of particular interest resulting in 36 miRNA-mRNA relations based on integrated data analysis. Of these 36 relations five miRNA-mRNA interactions have been validated previously. (TIF) [file pone.0135284.s006.tif]
