# Supplementary material for: Integrative Analysis of MicroRNA and mRNA Data Reveals an Orchestrated Function of MicroRNAs in Skeletal Myocyte Differentiation in Response to TNF-α or IGF1
Source: PLoS One. 2015 Aug 13;10(8):e0135284. doi: 10.1371/journal.pone.0135284 (PMC4536022; doi:10.1371/journal.pone.0135284)
Supplement: S2 Table — Target enrichment in (A) genes retrieved in signal transduction pathway associations by co-citation on the sentence level, as well as (B) MeSH disease terms (# genes (observed): number of genes of the input set which have the respective annotation; # genes (expected): number of genes expected with the respective annotation which is calculated based on the total number of genes with the respective annotation and the number of genes of the input set with the respective annotation; # genes (total): total number of genes with the respective annotation). Only the top 20 terms with p-values < 0.01 within the respective list were shown. (DOCX) [file pone.0135284.s008.docx]

**S2 Table. miRNA target expressions were enriched in distinct pathways.**

Target enrichment in **(A)** genes enriched in signal transduction pathway associations by co-citation on the sentence level, as well as **(B)** MeSH disease terms (# genes (observed): number of genes of the input set which have the respective annotation; # genes (expected): number of genes expected with the respective annotation which is calculated based on the total number of genes with the respective annotation and the number of genes of the input set with the respective annotation; # genes (total): total number of genes with the respective annotation). Only the top 20 terms with p-values < 0.01 within the respective list were shown. **(A)**

| **Pathway** | **P-value** | **# Genes (observed)** | **List of observed genes** |
| --- | --- | --- | --- |
| CYCLIN A2 | 2.70E-06 | 22 | Ccna2, Cdk1, Odc1, Cdc6, Ccnd1, Cdc25a, Mybl2, Wee1, Ccnb1, Ccnd2, Cdt1, Slbp, Csnk2a2, Trim28, Cdk6, Plk1, Creb1, Mki67, Chek1, Elf4, Mcm4, Cdkn1a |
| CELL DIVISION CYCLE 2, G1 TO S AND G2 TO M | 1.06E-05 | 38 | Ccna2, Elavl1, Cdk1, Cdc14b, Dab2, Cdc6, Cdc25a, Mcl1, Bcl2, Mre11a, Wee1, Ccnb1, Cdc14a, Rptor, Prc1, Slbp, Rcc1, Hmga1, Hmmr, Aurka, Cdc25b, Espl1, Cdk6, Plk1, Fbxo5, Kif2c, Kif11, Runx2, Hist1h3f, Chek1, Birc5, Nde1, Kif23, Npm1, Ncl, Runx1, Cdkn1a, Ccnb2 |
| CYCLIN B1 | 2.10E-05 | 19 | Ccna2, Cdk1, Ccnd1, Cdc25a, Bcl2, Wee1, Ccnb1, Prc1, Sod2, Aurka, Espl1, Atr, Plk1, Hist1h3f, Rps6ka1, Chek1, Birc5, Cdkn1a, Ccnb2 |
| CELL DIVISION CYCLE 25C | 2.25E-05 | 16 | Ccna2, Cdk1, Cdc25a, Wee1, Ccnb1, Atm, Cdc25b, Atr, Plk1, Hist1h3f, Brca1, Chek1, Plk3, Birc5, Npm1, Cdkn1a |
| MINICHROMOSOME MAINTENANCE COMPLEX | 3.17E-05 | 13 | Ccna2, Cdk1, Mcm6, Ccnd1, Mcm10, Atm, Dbf4, Atr, Cdc7, Emb, Mcm7, Chek1, Mcm4 |
| ATAXIA TELANGIECTASIA AND RAD3 RELATED | 3.88E-05 | 24 | Cdc6, Msh2, Aatf, Fhit, Rad9, Cdc25a, Mcl1, Mre11a, Smc1a, Ccnb1, Atm, Snip1, Mcph1, Fancd2, Clspn, Atr, Rad50, Plk1, Cdc7, Obfc1, Mdc1, Brca1, Topbp1, Chek1 |
| BREAST CANCER 1, EARLY ONSET | 1.72E-04 | 21 | Bard1, Msh2, Rad9, Cdc25a, Mre11a, Smc1a, Atm, Aurka, Mcph1, Fancd2, Clspn, Bmi1, Rad51, Atr, Brip1, Map3k3, Mdc1, Brca1, Topbp1, Chek1, Rnf168 |
| TUMOR PROTEIN P53 | 2.63E-04 | 71 | Bax, Bard1, Hoxa5, Pmaip1, Msh2, Apaf1, Nqo1, Aatf, Fhit, E2f3, Cdc25a, Ttk, Tbx3, Bcl2, Vrk1, Ppp1ca, Casp9, Rrm2, Kras, Smc1a, Eif4g1, Trp53inp1, Ccnb1, Bcl2l12, Atm, Gnl3, Psrc1, Pou2f1, Aurka, Irf5, Zmpste24, Tbp, Bmi1, Polh, Wisp1, Kat2b, Cryz, Hira, Strap, Atr, Plk1, Serpinb5, Sesn1, Twist1, Hnrnpc, Pms2, Bcl6, Ccne2, Wdr36, Txnrd1, Obfc1, Btg2, Brca1, Emb, Bok, Mki67, Taf1, Chek1, Plk3, Birc5, Eda2r, Usp7, Blm, Npm1, Gart, Eif5a, Prodh, Arid3a, Cdkn1a, Gtse1, Unc5b |
| PLATELET DERIVED GROWTH FACTOR RECEPTOR | 3.06E-04 | 33 | Vav2, Pdgfra, Flt1, Tnc, Csk, Gja1, Ptpn2, Jag1, Ank3, Abl2, Vcan, Mllt4, Etv6, Kitl, Sla, Rasa1, Vegfa, Acp1, Ywhag, S1pr1, Fn1, Dcbld2, Foxc2, Pld1, Lrp1, Csf1, Ptprj, Hhip, Nrp1, Cspg4, Shb, Sh2b2, Abl1 |
| EPHRIN | 5.31E-04 | 23 | Cbl, Efna5, Efnb1, Epha4, Cdh5, Fgfr1, Abl2, Adam19, Mllt4, Efnb2, Rgs3, Sla, Rasa1, Wwtr1, Acp1, Epha2, Efna1, Ephb4, Arf6, Nck2, Reln, Sp7, Abl1 |
| CYCLIN DEPENDENT KINASE | 5.96E-04 | 54 | Ccna2, Myh10, Cdk1, Cdc14b, Usp37, Cdc6, Efna5, Id2, Ccnd1, E2f3, Cdc25a, Mybl2, Cables1, Lin9, Cdk5r1, Wee1, Ccnb1, Ccnd2, Cdc14a, Rbl1, Cdt1, Slbp, Supt5h, Mapt, Hexim1, Cdc25b, Pold3, Cdk8, Sema3a, Cdk6, Uhrf2, Nefm, Cdk9, Sipa1l1, Cdc7, Ubtf, Lig1, Mpz, Ppm1a, Smurf1, E2f2, Mcm7, Mki67, Chek1, Klf6, Npm1, Mcm4, Reln, Cks1b, Runx1, Cdkn1a, Fen1, Far1, Prkar1a |
| FOCAL ADHESION KINASE 1 | 6.36E-04 | 39 | Capn2, Tnc, Actr2, Mme, Dusp22, Csk, Mgat5, Cttn, Rgnef, Mertk, Mbd2, Itgb3, Arhgef12, Itga4, Wisp1, Scara5, Tnk1, Ptk2b, Sema3a, Slc12a2, Plaur, Serpinb5, Epha2, Actn1, Cxcl12, Emp2, Tln1, Fn1, Slk, Ptk2, Itgb1, Dlc1, Src, Mapk11, Cspg4, Nck2, Shb, Spp1, Tgfb1i1 |
| CYCLIN E | 1.47E-03 | 16 | Ccna2, Cdk1, Odc1, Ccne1, Cdc6, Ccnd1, Cdc25a, Ccnb1, Ccnd2, Cebpb, Cdk6, Ccne2, Chek1, Npm1, Cdkn1a, Prkar1a |
| AURORA KINASE | 1.95E-03 | 19 | Mapre1, Cdk1, Tpx2, Rock2, Ccnb1, Cdc14a, Psrc1, Kpnb1, Aurka, Gsg2, Cdc25b, Plk1, Dtl, Kif2c, Cenpa, Kif2a, Hist1h3f, Brca1, Birc5 |
| RHOA RAS HOMOLOG | 3.39E-03 | 40 | Ezr, F2r, Procr, Vav2, Bves, Capn2, Tnc, Mkl1, Cfl1, Rock2, Icam1, Rgnef, Arhgdia, Sox9, Lingo1, Arhgap5, Smn1, Arhgef12, Akap13, Cdh13, Rhog, Plk1, Gdi1, Efna1, S1pr1, Fn1, Slk, Ptk2, Smurf1, Tjp2, Smarca4, Pld1, Gnaq, Dlc1, Diap3, Mprip, Ctgf, Hmgcr, Plxnb1, Tgfb1i1 |
| FANCONI ANEMIA COMPLEMENTATION GROUP COMPLEX | 5.65E-03 | 12 | Smc1a, Atm, Fancd2, Fancl, Atr, Brip1, Brca1, Usp1, Rad18, Chek1, C230052I12Rik, Blm |
| CYCLIN B2 | 6.66E-03 | 3 | Cdk1, Ccnb1, Ccnb2 |
| CYCLIN DEPENDENT KINASE INHIBITOR 1 | 7.79E-03 | 27 | Ccna2, Elavl1, Cdk1, Map3k5, Pmaip1, Ccnd1, Mcl1, Bcl2, Kras, Wee1, Ccnb1, Ccnd2, Pkd2, Atm, Xpo1, Bmi1, Strap, Cdk6, Uhmk1, Ccne2, Klf4, Calm1, Mki67, Chek1, Npm1, Hmgcr, Cdkn1a |
| WEE1 HOMOLOG | 8.17E-03 | 7 | Ccna2, Cdk1, Cables1, Wee1, Ccnb1, Chek1, Cdkn1a |
| TYROSINE PROTEIN KINASE SRC | 9.54E-03 | 58 | Ezr, G6pdx, F2r, Cbl, Asap1, Myh10, Vav2, Arrb1, Fhit, E2f3, Blk, Trip10, Hpgds, Cdh5, Csk, Rrm2, Gja1, Cttn, Lyn, Itgb3, Hck, Prkcd, Selp, Kcna5, Tnk2, Rap1b, Sla, Rasa1, Tnk1, Ptk2b, Tyro3, Fbxo5, Prkce, Ptpn6, Epha2, Actn1, Cxcl12, Med28, Pecam1, Fn1, Slk, Cckar, Ptk2, Pigr, Ceacam1, Itgb1, Shc2, Cnr1, Khdrbs1, Src, Nck2, Shb, Spp1, Egfr, Rhou, Plxnb1, Abl1, Adamts7 |

**(B)**

| **MeSH-Term** | **P-value** | **# Genes (observed)** | **# Genes (expected)** | **# Genes (total)** |
| --- | --- | --- | --- | --- |
| Neoplasms by Site | 3.85E-37 | 1673 | 1414.184123 | 10485 |
| Neoplasms | 9.93E-34 | 1855 | 1634.438074 | 12118 |
| Neoplasms, Glandular and Epithelial | 1.73E-32 | 1393 | 1136.47262 | 8426 |
| Neoplastic Processes | 2.39E-32 | 1093 | 839.7434767 | 6226 |
| Neoplasms by Histologic Type | 2.68E-32 | 1642 | 1399.887174 | 10379 |
| Carcinoma | 3.79E-31 | 1231 | 979.2061742 | 7260 |
| Breast Diseases | 5.11E-31 | 933 | 693.9415656 | 5145 |
| Breast Neoplasms | 5.28E-31 | 928 | 689.3557516 | 5111 |
| Skin Diseases | 2.10E-30 | 1204 | 955.7375965 | 7086 |
| Adenocarcinoma | 9.23E-30 | 1057 | 815.8702683 | 6049 |
| Neoplasms, Germ Cell and Embryonal | 9.80E-29 | 1056 | 819.2421904 | 6074 |
| Neuroectodermal Tumors | 1.44E-28 | 989 | 756.2546858 | 5607 |
| Neoplasms, Nerve Tissue | 2.99E-28 | 1001 | 768.9331128 | 5701 |
| Cell Transformation, Neoplastic | 2.49E-27 | 811 | 596.1558251 | 4420 |
| Digestive System Neoplasms | 1.11E-26 | 1164 | 932.9434032 | 6917 |
| Neoplasm Invasiveness | 1.20E-26 | 625 | 432.954796 | 3210 |
| Respiratory Tract Neoplasms | 3.20E-25 | 811 | 604.6530687 | 4483 |
| Urogenital Neoplasms | 7.99E-25 | 939 | 725.2330026 | 5377 |
| Colorectal Neoplasms | 1.28E-24 | 782 | 580.6449835 | 4305 |
| Gastrointestinal Neoplasms | 1.31E-24 | 942 | 729.0095553 | 5405 |
