# Supplementary material for: Integrative Analysis of MicroRNA and mRNA Data Reveals an Orchestrated Function of MicroRNAs in Skeletal Myocyte Differentiation in Response to TNF-α or IGF1
Source: PLoS One. 2015 Aug 13;10(8):e0135284. doi: 10.1371/journal.pone.0135284 (PMC4536022; doi:10.1371/journal.pone.0135284)
Supplement: S3 Table — Enrichment analysis of targeted transcription factors for signal transduction pathway associations by co-citation. (DOCX) [file pone.0135284.s009.docx]

**S3 Table. Pathway enrichment of targeted transcription factors.**

Enrichment analysis of targeted transcription factors for signal transduction pathway associations by co-citation.

| **Pathway** | **P-value** | **# Genes (observed)** | **List of observed genes** |
| --- | --- | --- | --- |
| TGF BETA | 1.17E-13 | 44 | Skil, Foxn1, Foxl2, Eomes, Dlx5, Smad9, Lhx2, Smad7, Id2, Kat2b, Cited2, Klf11, Foxc2, Foxd1, Foxc1, Dlx1, Smad6, Id1, Sox4, Tbx3, Strap, Ets1, Hivep2, Dlx3, Dlx2, Hmga2, Gli2, Runx1, Tbx20, Tgfb1i1, Ebf1, Bmi1, Klf10, Hivep3, Klf4, Sox9, Rlim, Klf6, Sp7, Nfya, Twist1, Runx2, Zeb1, Zfp521 |
| MOTHERS AGAINST DPP HOMOLOG | 1.46E-09 | 28 | Skil, Eomes, Dlx5, Smad9, Smad7, Id2, Kat2b, Cited2, Klf11, Foxc2, Smad6, Id1, Snip1, Strap, Ets1, Dlx2, Hmga2, Yap1, Runx1, Tbx20, Tgfb1i1, Klf10, Klf4, Sox9, Rlim, Sp7, Runx2, Zeb1 |
| RETINOBLASTOMA 1 | 9.17E-07 | 12 | Cdk9, Id2, Max, Ccnd1, Gmnn, E2f3, Dlx1, Rbl1, Dlx2, Taf1, E2f7, Zeb1 |
| WINGLESS TYPE | 1.24E-05 | 29 | Foxn1, Tle1, Foxl2, Atoh1, Cdx1, Hoxd9, Mbd2, Foxl1, Smarca4, Sox11, Gbx2, Foxc1, Irf6, Sox4, Chd8, Tbx3, Twist2, Ldb1, Sall1, Prdm5, Yap1, Tbl1x, Bmi1, Klf4, Sox9, Sp7, Hmga1, Runx2, Egr2 |
| NOTCH | 1.07E-04 | 18 | Foxn1, Tle1, Atoh1, Hif1an, Spen, Gata3, Dlx1, Id1, Irf6, Dlx2, Yap1, Runx1, Ebf1, Bmi1, Klf4, Sox9, Nr2f2, Zeb1 |
| CYCLIN D1 | 2.21E-04 | 11 | Ccnd1, Cdc25a, Mybl2, Id1, Pcgf2, Rbl1, Fosl1, Gli2, Taf1, Tbp, Klf6 |
| CYCLIN A2 | 2.80E-04 | 7 | Ccnd1, Cdc25a, Trim28, Mybl2, Creb1, Taf1, Tbp |
| HISTONE DEACETYLASE | 3.66E-04 | 8 | Pias2, Hdac7, Gata3, Ar, Sp3, Runx1, Tbl1x, Klf4 |
| FIBROBLAST GROWTH FACTOR | 3.83E-04 | 17 | Foxn1, Dlx5, Cdx1, Lhx2, Gbx2, Foxc1, Dlx1, Tbx3, Ets1, Irx2, Wwtr1, Dlx2, Sox9, Twist1, Runx2, Etv4, Egr2 |
| E2F TRANSCRIPTION FACTOR 1 | 4.08E-04 | 7 | Ncoa3, Ccnd1, E2f3, Myb, Topbp1, Mybl2, E2f2 |
| NUCLEAR RECEPTOR SUBFAMILY 2, GROUP F, MEMBER 1 | 7.07E-04 | 2 | Nr2f1, Nr2f2 |
| INDIAN HEDGEHOG | 1.05E-03 | 5 | Gli2, Sox9, Sp7, Runx2, Nr2f2 |
| CASEIN KINASE 2 | 1.33E-03 | 14 | Tle1, Cdk9, Max, Dek, Gmnn, Myb, Topbp1, Cbx1, Bdp1, Ubtf, Sp3, Taf1, Tbp, Hmga1 |
| SWI/SNF RELATED, MATRIX ASSOCIATED, ACTIN DEPENDENT REGULATOR OF CHROMATIN, SUBFAMILY A, MEMBER 4 (BRG1) | 1.95E-03 | 3 | Smarcd1, Smarca4, Cbx3 |
| FBJ MURINE OSTEOSARCOMA VIRAL ONCOGENE HOMOLOG B | 1.95E-03 | 3 | Fosb, Creb1, Fosl1 |
| CCAAT/ENHANCER BINDING PROTEIN (C/EBP), BETA | 2.66E-03 | 6 | Cdk9, Cebpb, Creb1, Atf6, Cebpa, Pou2f1 |
| TUMOR PROTEIN P53 | 3.36E-03 | 16 | Bcl6, Aatf, Kat2b, Brca1, E2f3, Irf5, Cdc25a, Tbx3, Hoxa5, Arid3a, Taf1, Tbp, Bmi1, E2f7, Twist1, Pou2f1 |
| NUCLEAR RECEPTOR SUBFAMILY 5, GROUP A, MEMBER 1 | 3.50E-03 | 5 | Foxl2, Cited2, Creb1, Sox9, Lhx9 |
| PARATHYROID HORMONE RELATED PROTEIN | 3.92E-03 | 7 | Gli2, Nr4a2, Sox9, Sp7, Elf4, Runx2, Zfp521 |
| ATAXIA TELANGIECTASIA AND RAD3 RELATED | 4.21E-03 | 6 | Aatf, Brca1, Cdc25a, Topbp1, Trim28, Snip1 |
| ATAXIA TELANGIECTASIA MUTATED | 4.21E-03 | 7 | Aatf, Brca1, Cdc25a, Topbp1, Trim28, Abl1, Elf4 |
| LARGE TUMOR SUPPRESSOR | 4.37E-03 | 3 | Foxl2, Wwtr1, Yap1 |
| ESTROGEN RECEPTOR | 4.68E-03 | 8 | Foxl2, Ncoa3, Kat2b, Brca1, Smarca4, Gata3, Ar, Nfatc4 |
| PROTOONCOGENE / PROTEIN KINASE PIM | 7.18E-03 | 5 | Myb, Cdc25a, Nfatc1, Ar, Fosl1 |
| P90 RIBOSOMAL PROTEIN S6 KINASE | 7.72E-03 | 7 | Kat2b, Cebpb, Smarca4, Creb1, Cbx3, Nfatc4, Klf6 |
| CYCLIN G2 | 9.88E-03 | 2 | Cdc25a, E2f2 |
| CYCLIN DEPENDENT KINASE | 9.93E-03 | 12 | Cdk9, Id2, Ccnd1, E2f3, Cdc25a, Mybl2, E2f2, Rbl1, Cdk8, Ubtf, Runx1, Klf6 |
