# Supplementary material for: Integrative Analysis of MicroRNA and mRNA Data Reveals an Orchestrated Function of MicroRNAs in Skeletal Myocyte Differentiation in Response to TNF-α or IGF1
Source: PLoS One. 2015 Aug 13;10(8):e0135284. doi: 10.1371/journal.pone.0135284 (PMC4536022; doi:10.1371/journal.pone.0135284)
Supplement: S4 Table — Differentiation- / TNFα- / IGF1-associated miRNAs potentially targeted inversely correlated genes which clustered by self-organizing tree algorithm (SOTA) analysis. Cluster analysis was performed for 0–72 h differentiation / treatment, however, only expression values for 24 h differentiation / treatment were depicted. MiRNA-mRNA relations were depicted for: (A) cluster of genes which were up-regulated in very early differentiation, (B) cluster of genes which were up-regulated in later differentiation, (C) genes which were down regulated during very early or later differentiation, (D) genes which were down-regulated later during differentiation. (DOCX) [file pone.0135284.s010.docx]

**S4 Table. Gene expression clusters and their targeting miRNA-relations.**

Differentiation- / TNFα- / IGF1-associated miRNAs potentially targeted inversely correlated genes which clustered by self-organizing tree algorithm (SOTA) analysis. Cluster analysis was performed for 0 – 72 h differentiation / treatment, however, only expression values for 24 h differentiation / treatment were depicted. MiRNA-mRNA relations were depicted for: **(A)** cluster of genes which were up-regulated in very early differentiation, **(B)** cluster of genes which were up-regulated in later differentiation, **(C)** genes which were down regulated during very early or later differentiation, **(D)** genes which were down-regulated later during differentiation.

**(A)**

| **gene symbol** | **targeting miRNAs** |
| --- | --- |
| A930001N09Rik | mmu-miR-155 |
| Ablim1 | mmu-miR-155 |
| Adamts4 | mmu-miR-155 |
| Adamts5 | mmu-miR-155 |
| Adck3 | mmu-miR-155 |
| Arpp21 | mmu-miR-155 |
| Bcl6 | mmu-miR-155 |
| Bnc2 | mmu-miR-155, mmu-miR-29b |
| Car5b | mmu-miR-155 |
| Casp12 | mmu-miR-155 |
| Ctso | mmu-miR-155 |
| Dync1i1 | mmu-miR-155 |
| E2f2 | mmu-miR-155 |
| Fgfr4 | mmu-miR-155 |
| Frmd4b | mmu-miR-155 |
| Gabrb2 | mmu-miR-155 |
| Hmgcs1 | mmu-miR-155, mmu-miR-29b |
| Hspa4l | mmu-miR-155, mmu-miR-29b |
| Htra3 | mmu-miR-155 |
| Idh1 | mmu-miR-155 |
| Idi1 | mmu-miR-155 |
| Inadl | mmu-miR-155 |
| Itga4 | mmu-miR-155 |
| Lphn2 | mmu-miR-155 |
| Lrrn1 | mmu-miR-155 |
| Mfsd2a | mmu-miR-155 |
| Ogn | mmu-miR-155 |
| Pak3 | mmu-miR-155 |
| Pdlim3 | mmu-miR-155 |
| Sema3c | mmu-miR-155 |
| Sema5a | mmu-miR-155, mmu-miR-29b |
| Sgcb | mmu-miR-155 |
| Sort1 | mmu-miR-155 |
| Srebf2 | mmu-miR-155, mmu-miR-29b |
| Stc1 | mmu-miR-155 |
| Trp53inp1 | mmu-miR-155 |
| Vwa5a | mmu-miR-155 |
| Ypel3 | mmu-miR-155 |
| Zeb1 | mmu-miR-155 |
| Zfp238 | mmu-miR-155, mmu-miR-29b |
| Zfp346 | mmu-miR-155 |
| Ccdc80 | mmu-miR-29b |
| Ccdc88c | mmu-miR-29b |
| Cdkn1a | mmu-miR-29b |
| Chrnd | mmu-miR-29b |
| Daam2 | mmu-miR-29b |
| Eda2r | mmu-miR-29b |
| Frmpd1 | mmu-miR-29b |
| Gramd1b | mmu-miR-29b |
| Insig1 | mmu-miR-29b |
| Klhdc10 | mmu-miR-29b |
| Mc4r | mmu-miR-29b |
| Mfap5 | mmu-miR-29b |
| Nrp2 | mmu-miR-29b |
| Pde1a | mmu-miR-29b |
| Ppfia4 | mmu-miR-29b |
| Scn7a | mmu-miR-29b |
| Tmem8c | mmu-miR-29b |
| AI464131 | mmu-miR-202-3p |
| Jam3 | mmu-miR-202-3p |

**(B)**

| **symbol** | **targeting.miRNAs** |
| --- | --- |
| Atp1b1 | mmu-miR-335-3p |
| Klf4 | mmu-miR-206 |
| Pkp1 | mmu-miR-133a, mmu-miR-133b, mmu-miR-532-5p |
| Abcc9 | mmu-miR-155 |
| Acta1 | mmu-miR-155 |
| Baiap2l1 | mmu-miR-155 |
| Cap2 | mmu-miR-155 |
| F2rl1 | mmu-miR-155 |
| Fn3k | mmu-miR-155 |
| Itgb6 | mmu-miR-155 |
| Kbtbd12 | mmu-miR-155 |
| Kcnj2 | mmu-miR-155 |
| Lmo7 | mmu-miR-155 |
| Myh8 | mmu-miR-155 |
| Mylk4 | mmu-miR-155 |
| Myoz2 | mmu-miR-155 |
| Mypn | mmu-miR-155 |
| Pfkm | mmu-miR-155 |
| Pln | mmu-miR-155 |
| Slc16a2 | mmu-miR-155 |
| Tspan7 | mmu-miR-155 |
| Txlnb | mmu-miR-155 |
| Sox11 | mmu-miR-351 |
| C1qtnf3 | mmu-miR-29b |
| Csdc2 | mmu-miR-29b |
| Fras1 | mmu-miR-29b |
| Fsd2 | mmu-miR-29b |
| Gja5 | mmu-miR-29b |
| Mtss1l | mmu-miR-29b |
| Myoz1 | mmu-miR-29b |
| Smtnl2 | mmu-miR-29b |
| Spry1 | mmu-miR-29b |
| Sv2b | mmu-miR-29b |
| Prelid2 | mmu-miR-378a-5p |

**(C)**

| **symbol** | **targeting.miRNAs** |
| --- | --- |
| Abcc1 | mmu-miR-133a, mmu-miR-133b, mmu-miR-335-3p, mmu-miR-335-5p |
| Adcy8 | mmu-miR-335-3p |
| Alcam | mmu-miR-206, mmu-miR-335-3p, mmu-miR-483-3p |
| Atp10d | mmu-miR-206, mmu-miR-335-3p, mmu-miR-532-3p, mmu-miR-532-5p |
| Cd24a | mmu-miR-335-3p |
| Chaf1b | mmu-miR-335-3p |
| Cxcl12 | mmu-miR-133b, mmu-miR-335-3p, mmu-miR-335-5p, mmu-miR-351 |
| Dlx2 | mmu-miR-335-3p |
| Dock8 | mmu-miR-133a, mmu-miR-133b, mmu-miR-206, mmu-miR-322, mmu-miR-335-3p, mmu-miR-351 |
| Dtl | mmu-miR-335-3p |
| Ezr | mmu-miR-335-3p |
| Fgf7 | mmu-miR-322, mmu-miR-322-3p, mmu-miR-335-3p |
| Gas6 | mmu-miR-335-3p |
| Gja1 | mmu-miR-322-3p, mmu-miR-335-3p |
| Gnb4 | mmu-miR-335-3p, mmu-miR-335-5p, mmu-miR-483-3p |
| Havcr2 | mmu-miR-335-3p, mmu-miR-532-3p |
| Hells | mmu-miR-335-3p |
| Hmga2 | mmu-miR-322, mmu-miR-322-3p, mmu-miR-335-3p, mmu-miR-335-5p, mmu-miR-532-3p |
| Hunk | mmu-miR-335-3p, mmu-miR-532-5p |
| Id1 | mmu-miR-335-3p |
| Igsf11 | mmu-miR-335-3p, mmu-miR-335-5p |
| Igsf5 | mmu-miR-335-3p, mmu-miR-351 |
| Il18rap | mmu-miR-133b, mmu-miR-335-3p, mmu-miR-351, mmu-miR-378a-5p |
| Itga6 | mmu-miR-206, mmu-miR-335-3p, mmu-miR-532-3p |
| Lrp8 | mmu-miR-335-3p, mmu-miR-335-5p |
| Mthfd2 | mmu-miR-335-3p |
| Neto2 | mmu-miR-322, mmu-miR-322-3p, mmu-miR-335-3p, mmu-miR-335-5p |
| Pappa | mmu-miR-335-3p |
| Pde3b | mmu-miR-335-3p |
| Pla2g4a | mmu-miR-206, mmu-miR-335-3p |
| Prr9 | mmu-miR-335-3p, mmu-miR-335-5p |
| Ptgs2 | mmu-miR-335-3p |
| Rapgef4 | mmu-miR-335-3p, mmu-miR-378a-5p |
| Rasl11a | mmu-miR-335-3p, mmu-miR-532-5p |
| Relt | mmu-miR-133a, mmu-miR-133b, mmu-miR-335-3p |
| Ret | mmu-miR-206, mmu-miR-335-3p |
| Rrm2 | mmu-miR-335-3p, mmu-miR-335-5p |
| Slc7a11 | mmu-miR-322-3p, mmu-miR-335-3p, mmu-miR-532-5p |
| Slfn9 | mmu-miR-335-3p |
| Smoc2 | mmu-miR-335-3p, mmu-miR-335-5p |
| Timp3 | mmu-miR-206, mmu-miR-335-3p |
| Unc5b | mmu-miR-335-3p |
| Wnt5a | mmu-miR-335-3p |
| Adam12 | mmu-miR-206, mmu-miR-532-5p |
| Arrdc3 | mmu-miR-206 |
| Ccnd1 | mmu-miR-206, mmu-miR-322, mmu-miR-503 |
| Chst11 | mmu-miR-206, mmu-miR-532-5p |
| Fbxo48 | mmu-miR-206 |
| Glrp1 | mmu-miR-206, mmu-miR-335-5p |
| Jag1 | mmu-miR-206 |
| Lce1g | mmu-miR-206 |
| Mcm10 | mmu-miR-206 |
| Nr4a2 | mmu-miR-206 |
| Sema6d | mmu-miR-206 |
| Slc16a13 | mmu-miR-206, mmu-miR-322-3p, mmu-miR-483-3p |
| Slc1a4 | mmu-miR-206 |
| Smad9 | mmu-miR-206 |
| Vegfa | mmu-miR-206, mmu-miR-322, mmu-miR-351, mmu-miR-532-3p |
| Ccbe1 | mmu-miR-351, mmu-miR-483-3p |
| Efna5 | mmu-miR-378a-5p, mmu-miR-483-3p, mmu-miR-532-3p |
| Sp7 | mmu-miR-483-3p |
| Ubash3b | mmu-miR-351, mmu-miR-483-3p, mmu-miR-542-5p |
| Ak4 | mmu-miR-322, mmu-miR-503 |
| Bcat1 | mmu-miR-322, mmu-miR-335-5p, mmu-miR-351, mmu-miR-503 |
| Cdca7 | mmu-miR-139-5p, mmu-miR-322 |
| Clspn | mmu-miR-322, mmu-miR-532-3p |
| Cx3cl1 | mmu-miR-322 |
| Egln3 | mmu-miR-322, mmu-miR-322-3p, mmu-miR-450a-5p |
| Hk2 | mmu-miR-322, mmu-miR-351 |
| Id2 | mmu-miR-322 |
| Kcnn4 | mmu-miR-322, mmu-miR-532-5p |
| Nmnat2 | mmu-miR-322, mmu-miR-335-5p |
| Parvb | mmu-miR-322 |
| Smad7 | mmu-miR-322, mmu-miR-322-3p |
| Snhg1 | mmu-miR-322, mmu-miR-351 |
| Syt12 | mmu-miR-322 |
| Dhrs3 | mmu-miR-532-3p |
| Greb1l | mmu-miR-532-3p, mmu-miR-532-5p |
| Hbegf | mmu-miR-378a-5p, mmu-miR-532-3p |
| Irf5 | mmu-miR-532-3p |
| Pgf | mmu-miR-378a-5p, mmu-miR-532-3p, mmu-miR-532-5p |
| Cpm | mmu-miR-532-5p |
| Exo1 | mmu-miR-133a, mmu-miR-133b, mmu-miR-532-5p |
| Npr3 | mmu-miR-532-5p |
| Optn | mmu-miR-335-5p, mmu-miR-532-5p |
| Atoh8 | mmu-miR-351 |
| Cpne2 | mmu-miR-335-5p, mmu-miR-351 |
| Ctgf | mmu-miR-351 |
| Fam78b | mmu-miR-133b, mmu-miR-351 |
| Foxq1 | mmu-miR-351 |
| Gpr114 | mmu-miR-351 |
| Prkg2 | mmu-miR-351 |
| C3ar1 | mmu-miR-335-5p |
| Etv4 | mmu-miR-335-5p |
| Kazald1 | mmu-miR-335-5p |
| Nipal1 | mmu-miR-335-5p |
| Wdhd1 | mmu-miR-335-5p |
| Dlx1 | mmu-miR-133a, mmu-miR-133b |
| Ermp1 | mmu-miR-133a |
| Foxc2 | mmu-miR-133a, mmu-miR-133b |
| Gpr97 | mmu-miR-133a, mmu-miR-133b |
| Smad6 | mmu-miR-133a, mmu-miR-133b |
| Twist2 | mmu-miR-133a, mmu-miR-133b |
| Ereg | mmu-miR-322-3p |
| Lgr6 | mmu-miR-322-3p |
| Mybl2 | mmu-miR-322-3p |
| Pde8a | mmu-miR-322-3p |
| Serpinb2 | mmu-miR-322-3p |
| Serpine1 | mmu-miR-322-3p |
| Slc7a1 | mmu-miR-322-3p |
| Cdt1 | mmu-miR-503 |
| Fam111a | mmu-miR-378a-5p |
| Dusp9 | mmu-miR-542-5p |

**(D)**

| **symbol** | **targeting.miRNAs** |
| --- | --- |
| Bend6 | mmu-miR-335-3p, mmu-miR-335-5p, mmu-miR-532-5p |
| Car8 | mmu-miR-335-3p |
| Cobll1 | mmu-miR-206, mmu-miR-335-3p, mmu-miR-378a-5p, mmu-miR-483-3p, mmu-miR-532-5p |
| Espl1 | mmu-miR-335-3p |
| Fam19a5 | mmu-miR-206, mmu-miR-335-3p |
| Fbxo5 | mmu-miR-335-3p |
| Gins1 | mmu-miR-335-3p |
| Hgf | mmu-miR-206, mmu-miR-322, mmu-miR-335-3p |
| Kif18a | mmu-miR-335-3p |
| Adamts3 | mmu-miR-206, mmu-miR-322, mmu-miR-503 |
| Chek1 | mmu-miR-206, mmu-miR-322, mmu-miR-322-3p, mmu-miR-503 |
| Nefl | mmu-miR-206 |
| Pdk4 | mmu-miR-322, mmu-miR-532-5p |
| Plaur | mmu-miR-322 |
| Abcc4 | mmu-miR-351 |
| Nefm | mmu-miR-351, mmu-miR-450a-5p |
| Nqo1 | mmu-miR-351 |
| Podxl | mmu-miR-351 |
| Txnrd1 | mmu-miR-351 |
| Srxn1 | mmu-miR-133a, mmu-miR-133b |
| Stac2 | mmu-miR-133a, mmu-miR-133b |
| Bard1 | mmu-miR-322-3p |
| Eme1 | mmu-miR-322-3p |
| Prr11 | mmu-miR-322-3p, mmu-miR-503 |
| Rad54l | mmu-miR-322-3p |
| Trip13 | mmu-miR-322-3p |
| Brca1 | mmu-miR-503 |
