# Supplementary material for: Integrative Analysis of MicroRNA and mRNA Data Reveals an Orchestrated Function of MicroRNAs in Skeletal Myocyte Differentiation in Response to TNF-α or IGF1
Source: PLoS One. 2015 Aug 13;10(8):e0135284. doi: 10.1371/journal.pone.0135284 (PMC4536022; doi:10.1371/journal.pone.0135284)
Supplement: S5 Table — SOTA analysis of gene expression data of skeletal myoblast differentiation and TNF-α and IGF1 treatment (0–72 h) revealed clusters of gene sets which were targeted by differentiation-associated miRNAs. Scaled expressions of miRNA-mRNA relations based on integrative analysis were depicted for 24 h differentiation / treatment. Clustered targets were retrieved in signal transduction pathway associations or in GO term ‘biological processes’: (A), (B) cluster of genes which were up-regulated in very early differentiation, (C), (D) cluster of genes which were up-regulated in later differentiation, (E), (F) genes which were down regulated during very early or later differentiation, (G), (H) genes which were down-regulated later during differentiation. (DOCX) [file pone.0135284.s011.docx]

**S5 Table. Enrichment of clusters of miRNA target expressions in gene ontology terms and pathways.**

SOTA analysis of gene expression data of skeletal myoblast differentiation and TNF-α and IGF1 treatment (0 – 72 h) revealed clusters of gene sets which were targeted by differentiation-associated miRNAs. Scaled expressions of miRNA-mRNA relations based on integrative analysis were depicted for 24 h differentiation / treatment. Clustered targets were enriched in signal transduction pathway associations or in GO term ‘biological processes’: **(A)**, **(B)** cluster of genes which were up-regulated in very early differentiation, **(C)**, **(D)** cluster of genes which were up-regulated in later differentiation, **(E)**, **(F)** genes which were down regulated during very early or later differentiation, **(G)**, **(H)** genes which were down-regulated later during differentiation.

**(A)**

| **Pathway** | **P-value** | **# Genes (observed)** | **List of observed genes** |
| --- | --- | --- | --- |
| SEMAPHORIN | 2.33E-03 | 3 | Nrp2, Sema3c, Sema5a |
| CANNABINOID RECEPTOR 1 (BRAIN) | 6.24E-03 | 2 | Cnr1, Mc4r |
| ADENYLATE CYCLASE | 9.72E-03 | 3 | Cnr1, Stc1, Arpp21 |

**(B)**

| **GO-Term** | **P-value** | **# Genes (observed)** | **# Genes (expected)** | **# Genes (total)** |
| --- | --- | --- | --- | --- |
| cellular process | 9.31E-08 | 54 | 34.39479744 | 13009 |
| steroid biosynthetic process | 8.91E-07 | 6 | 0.325202559 | 123 |
| steroid metabolic process | 2.48E-06 | 7 | 0.608102345 | 230 |
| cholesterol biosynthetic process | 2.74E-06 | 4 | 0.09782516 | 37 |
| skeletal muscle tissue development | 4.27E-06 | 6 | 0.425671642 | 161 |
| sterol biosynthetic process | 5.06E-06 | 4 | 0.113688699 | 43 |
| tissue development | 5.28E-06 | 13 | 2.932110874 | 1109 |
| cholesterol metabolic process | 5.29E-06 | 5 | 0.251172708 | 95 |
| skeletal muscle organ development | 5.45E-06 | 6 | 0.444179104 | 168 |
| sterol metabolic process | 7.50E-06 | 5 | 0.269680171 | 102 |
| programmed cell death | 1.31E-05 | 14 | 3.696204691 | 1398 |
| cell death | 2.26E-05 | 14 | 3.881279318 | 1468 |
| death | 2.41E-05 | 14 | 3.902430704 | 1476 |
| system development | 2.50E-05 | 20 | 7.511385928 | 2841 |
| isoprenoid biosynthetic process | 3.88E-05 | 3 | 0.066098081 | 25 |
| apoptotic process | 5.36E-05 | 13 | 3.651257996 | 1381 |
| organ development | 8.44E-05 | 16 | 5.565458422 | 2105 |
| muscle structure development | 8.61E-05 | 7 | 1.054925373 | 399 |
| blood vessel remodeling | 9.07E-05 | 3 | 0.087249467 | 33 |
| cell development | 9.19E-05 | 12 | 3.310191898 | 1252 |

**(C)**

| **Pathway** | **P-value** | **# Genes (observed)** | **List of observed genes** |
| --- | --- | --- | --- |
| CALCINEURIN (PROTEIN PHOSPHATASE 3) | 9.92E-06 | 5 | Myoz1, Abcc9, Pln, Myoz2, Kcnj2 |
| PROTEIN KINASE A | 3.10E-03 | 5 | Abcc9, Pln, Gja5, C1qtnf3, Kcnj2 |

**(D)**

| **GO-Term** | **P-value** | **# Genes (observed)** | **# Genes (expected)** | **# Genes (total)** |
| --- | --- | --- | --- | --- |
| regulation of ERK1 and ERK2 cascade | 1.49E-05 | 4 | 0.15010661 | 110 |
| negative regulation of chemokine production | 2.70E-05 | 2 | 0.008187633 | 6 |
| ERK1 and ERK2 cascade | 2.71E-05 | 4 | 0.17466951 | 128 |
| chemokine (C-X-C motif) ligand 2 production | 5.03E-05 | 2 | 0.010916844 | 8 |
| negative regulation of MAPK cascade | 1.19E-04 | 3 | 0.095522388 | 70 |
| transmembrane transport | 3.25E-04 | 7 | 1.360511727 | 997 |
| negative regulation of ERK1 and ERK2 cascade | 3.38E-04 | 2 | 0.027292111 | 20 |
| negative regulation of intracellular protein kinase cascade | 4.39E-04 | 3 | 0.148742004 | 109 |
| transport | 5.24E-04 | 12 | 4.268486141 | 3128 |
| negative regulation of response to cytokine stimulus | 5.31E-04 | 2 | 0.034115139 | 25 |
| establishment of localization | 6.19E-04 | 12 | 4.344904051 | 3184 |
| regulation of MAPK cascade | 7.95E-04 | 4 | 0.421663113 | 309 |
| cellular potassium ion transport | 8.19E-04 | 3 | 0.184221748 | 135 |
| potassium ion transmembrane transport | 8.19E-04 | 3 | 0.184221748 | 135 |
| cellular component assembly | 9.14E-04 | 7 | 1.617057569 | 1185 |
| localization | 9.64E-04 | 13 | 5.252366738 | 3849 |
| regulation of interleukin-8 production | 9.85E-04 | 2 | 0.046396588 | 34 |
| myofibril assembly | 1.04E-03 | 2 | 0.047761194 | 35 |
| interleukin-8 production | 1.04E-03 | 2 | 0.047761194 | 35 |
| filopodium assembly | 1.23E-03 | 2 | 0.051855011 | 38 |

**(E)**

| **Pathway** | **P-value** | **# Genes (observed)** | **List of observed genes** |
| --- | --- | --- | --- |
| MOTHERS AGAINST DPP HOMOLOG | 7.59E-05 | 13 | Foxc2, Atoh8, Smad9, Smad6, Id1, Smad7, Id2, Dlx2, Hmga2, Ctgf, Sp7, Timp3, Serpine1 |
| HYPOXIA INDUCIBLE FACTOR 1, ALPHA SUBUNIT (BASIC HELIX LOOP HELIX TRANSCRIPTION FACTOR) | 2.96E-04 | 7 | Egln3, Cxcl12, Id1, Abcc1, Vegfa, Ptgs2, Serpine1 |
| PARATHYROID HORMONE RELATED PROTEIN | 4.30E-04 | 6 | Gja1, Ctgf, Sp7, Nr4a2, Ezr, Jag1 |
| TGF BETA | 4.56E-04 | 16 | Adam12, Foxc2, Atoh8, Dlx1, Smad9, Smad6, Id1, Smad7, Id2, Dlx2, Hmga2, Ctgf, Sp7, Timp3, Dusp9, Serpine1 |
| VERY LOW DENSITY LIPOPROTEIN RECEPTOR | 7.03E-04 | 3 | Lrp8, Serpinb2, Serpine1 |
| MATRIX METALLOPROTEINASE | 1.94E-03 | 8 | Adam12, Wnt5a, Cxcl12, Ctgf, Timp3, Vegfa, Etv4, Ptgs2 |
| PEROXISOME PROLIFERATOR ACTIVATED RECEPTOR DELTA | 4.49E-03 | 3 | Pla2g4a, Nr4a2, Ptgs2 |
| INTEGRIN LINKED KINASE | 6.29E-03 | 4 | Cxcl12, Fgf7, Ctgf, Parvb |
| THROMBOSPONDIN 1 | 8.83E-03 | 3 | Ctgf, Ccnd1, Vegfa |

**(F)**

| **GO-Term** | **P-value** | **# Genes (observed)** | **# Genes (expected)** | **# Genes (total)** |
| --- | --- | --- | --- | --- |
| system development | 7.29E-16 | 47 | 13.32665245 | 2841 |
| anatomical structure morphogenesis | 8.77E-16 | 37 | 8.049466951 | 1716 |
| anatomical structure development | 5.04E-14 | 48 | 15.51257996 | 3307 |
| developmental process | 7.69E-14 | 51 | 17.64690832 | 3762 |
| multicellular organismal development | 1.10E-13 | 48 | 15.8315565 | 3375 |
| blood vessel morphogenesis | 4.10E-13 | 18 | 1.857569296 | 396 |
| locomotion | 8.42E-13 | 25 | 4.334328358 | 924 |
| negative regulation of cellular process | 2.54E-12 | 39 | 11.55820896 | 2464 |
| blood vessel development | 4.06E-12 | 18 | 2.129637527 | 454 |
| regulation of developmental process | 5.24E-12 | 28 | 6.032409382 | 1286 |
| regulation of cell proliferation | 8.42E-12 | 25 | 4.817484009 | 1027 |
| vasculature development | 9.87E-12 | 18 | 2.246908316 | 479 |
| regulation of biological process | 1.17E-11 | 75 | 40.02686567 | 8533 |
| cell differentiation | 1.18E-11 | 37 | 10.97185501 | 2339 |
| cell migration | 1.54E-11 | 21 | 3.3445629 | 713 |
| positive regulation of cellular process | 1.56E-11 | 40 | 12.86226013 | 2742 |
| cellular developmental process | 1.62E-11 | 38 | 11.67547974 | 2489 |
| cell proliferation | 1.97E-11 | 27 | 5.910447761 | 1260 |
| angiogenesis | 2.24E-11 | 15 | 1.47761194 | 315 |
| positive regulation of cell migration | 3.44E-11 | 13 | 1.027292111 | 219 |

**(G)**

| **Pathway** | **P-value** | **# Genes (observed)** | **List of observed genes** |
| --- | --- | --- | --- |
| NUCLEAR FACTOR (ERYTHROID DERIVED 2) LIKE 2 | 1.46E-04 | 3 | Nqo1, Txnrd1, Srxn1 |
| BREAST CANCER 1, EARLY ONSET | 4.20E-04 | 3 | Bard1, Chek1, Brca1 |
| TUMOR PROTEIN P53 | 6.36E-04 | 5 | Nqo1, Txnrd1, Bard1, Chek1, Brca1 |
| BREAST CANCER 2, EARLY ONSET | 1.00E-03 | 2 | Chek1, Brca1 |
| POLO LIKE KINASE 1 | 1.60E-03 | 3 | Fbxo5, Chek1, Espl1 |
| CELL DIVISION CYCLE 25C | 2.80E-03 | 2 | Chek1, Brca1 |
| FANCONI ANEMIA COMPLEMENTATION GROUP COMPLEX | 3.39E-03 | 2 | Chek1, Brca1 |
| CELL DIVISION CYCLE 2, G1 TO S AND G2 TO M | 3.56E-03 | 3 | Fbxo5, Chek1, Espl1 |
| CYCLIN B1 | 5.71E-03 | 2 | Chek1, Espl1 |

**(H)**

| **GO-Term** | **P-value** | **# Genes (observed)** | **# Genes (expected)** | **# Genes (total)** |
| --- | --- | --- | --- | --- |
| microtubule cytoskeleton organization | 9.27E-09 | 7 | 0.282089552 | 245 |
| microtubule-based process | 8.42E-08 | 7 | 0.389168443 | 338 |
| DNA metabolic process | 7.71E-07 | 8 | 0.80021322 | 695 |
| DNA recombination | 8.49E-07 | 5 | 0.178464819 | 155 |
| regulation of histone H3-K9 acetylation | 1.28E-06 | 2 | 0.002302772 | 2 |
| histone H3-K9 acetylation | 1.28E-06 | 2 | 0.002302772 | 2 |
| DNA repair | 1.28E-06 | 6 | 0.361535181 | 314 |
| double-strand break repair | 2.52E-06 | 4 | 0.096716418 | 84 |
| cellular response to stress | 3.50E-06 | 8 | 0.979829424 | 851 |
| meiotic spindle organization | 3.83E-06 | 2 | 0.003454158 | 3 |
| female meiosis I | 7.65E-06 | 2 | 0.004605544 | 4 |
| intermediate filament bundle assembly | 7.65E-06 | 2 | 0.004605544 | 4 |
| regulation of axon diameter | 7.65E-06 | 2 | 0.004605544 | 4 |
| cytoskeleton organization | 8.44E-06 | 7 | 0.776034115 | 674 |
| response to DNA damage stimulus | 1.07E-05 | 6 | 0.522729211 | 454 |
| recombinational repair | 1.12E-05 | 3 | 0.043752665 | 38 |
| double-strand break repair via homologous recombination | 1.12E-05 | 3 | 0.043752665 | 38 |
| protein K6-linked ubiquitination | 1.91E-05 | 2 | 0.006908316 | 6 |
| regulation of cell projection size | 1.91E-05 | 2 | 0.006908316 | 6 |
| response to stress | 3.00E-05 | 10 | 2.207206823 | 1917 |
