# Supplementary material for: Integrative Analysis of MicroRNA and mRNA Data Reveals an Orchestrated Function of MicroRNAs in Skeletal Myocyte Differentiation in Response to TNF-α or IGF1
Source: PLoS One. 2015 Aug 13;10(8):e0135284. doi: 10.1371/journal.pone.0135284 (PMC4536022; doi:10.1371/journal.pone.0135284)
Supplement: S6 Table — Enrichment analysis of signal transduction pathway associations of (A) miR-206-3p, (B) miR-322-3p, (C) miR-322-5p, (D) miR-335-3p, (E) miR-335-5p, (F) miR-351-5p, (G) miR-503-5p, (H) miR-133a-3p/miR-133b-3p, (I) miR-155-5p. (DOCX) [file pone.0135284.s012.docx]

**S6 Table. Pathway enrichment of targets of selected miRNAs.**

Enrichment analysis of signal transduction pathway associations of **(A)** miR-206-3p, **(B)** miR-322-3p, **(C)** miR-322-5p, **(D)** miR-335-3p, **(E)** miR-335-5p, **(F)** miR-351-5p, **(G)** miR-503-5p, **(H)** miR-133a-3p/miR-133b-3p, **(I)** miR-155-5p.

**(A)** miR-206-3p

| **Pathway** | **P-value** | **# Genes (observed)** | **List of observed genes** |
| --- | --- | --- | --- |
| SERINE/ARGININE SPLICING FACTOR PROTEIN KINASE | 1.14E-04 | 4 | Srsf1, Csnk2a2, Ccnd1, Slc39a14 |
| SEMAPHORIN | 1.98E-03 | 5 | Sema6d, Vegfa, Capn2, Plxna4, Arf6 |
| TGF BETA | 2.38E-03 | 19 | Vcan, Twsg1, Fbln2, Alpl, Crim1, Smad9, Gli2, Timp3, Tgfb1i1, Chsy1, Nup214, Cd109, Adam12, Klf4, Sox9, Fstl1, Tbx3, Runx2, Arhgap31 |
| CYCLIN A2 | 2.39E-03 | 5 | Csnk2a2, Ccnd1, Chek1, Cdc6, Wee1 |
| PARATHYROID HORMONE RELATED PROTEIN | 3.32E-03 | 6 | Gli2, Nr4a2, Jag1, Pdgfa, Sox9, Runx2 |
| FOCAL ADHESION KINASE 1 | 5.93E-03 | 8 | Itgb3, Hmmr, Mertk, Tgfb1i1, Capn2, Mgat5, Hgf, Dlc1 |
| EPH RECEPTOR | 6.21E-03 | 5 | Rasa1, Efna1, Rgs3, Arf6, Cbl |
| WEE1 HOMOLOG | 6.31E-03 | 3 | Csnk2a2, Chek1, Wee1 |
| CAVEOLIN 1 | 9.20E-03 | 5 | Vegfa, Mgat5, F3, Prkce, Arf6 |
| CHOLINERGIC RECEPTOR, MUSCARINIC | 9.40E-03 | 3 | Rgs4, Rasa1, Pla2g4a |
| MINICHROMOSOME MAINTENANCE COMPLEX | 9.40E-03 | 3 | Mcm10, Ccnd1, Chek1 |

**(B)** miR-322-3p

| **Pathway** | **P-value** | **# Genes (observed)** | **List of observed genes** |
| --- | --- | --- | --- |
| BREAST CANCER 1, EARLY ONSET | 4.87E-07 | 10 | Mcph1, Cdc25a, Topbp1, Fancd2, Rad51, Mre11a, Bard1, Atr, Chek1, Brip1 |
| ATAXIA TELANGIECTASIA AND RAD3 RELATED | 5.43E-06 | 9 | Blm, Mcph1, Cdc25a, Topbp1, Fancd2, Snip1, Mre11a, Atr, Chek1 |
| FANCONI ANEMIA COMPLEMENTATION GROUP COMPLEX | 7.33E-06 | 7 | Blm, Topbp1, Fancd2, Atr, Usp1, Chek1, Brip1 |
| CELL DIVISION CYCLE 2, G1 TO S AND G2 TO M | 1.52E-03 | 9 | Hmmr, Npm1, Cdc25a, Elavl1, Mre11a, Chek1, Cdc14b, Slbp, Hmga1 |
| VERY LOW DENSITY LIPOPROTEIN RECEPTOR | 3.56E-03 | 3 | Lrp1, Serpinb2, Serpine1 |
| CELL DIVISION CYCLE 25C | 4.55E-03 | 4 | Npm1, Cdc25a, Atr, Chek1 |
| TUMOR PROTEIN P53 | 5.04E-03 | 14 | Ywhag, Blm, Npm1, Cdc25a, Wdr36, Rad51, Gnl3, Ppm1l, Bard1, Atr, Polh, Kras, Chek1, E2f7 |
| WISKOTT ALDRICH SYNDROME LIKE | 6.32E-03 | 3 | Actr2, Wipf1, Ptk2 |
| BREAST CANCER 2, EARLY ONSET | 7.46E-03 | 3 | Fancd2, Chek1, Brip1 |
| ATAXIA TELANGIECTASIA MUTATED | 8.09E-03 | 6 | Cdc25a, Topbp1, Fancd2, Mre11a, Atr, Chek1 |
| CYCLIN DEPENDENT KINASE | 8.39E-03 | 11 | Npm1, Cdc25a, Mybl2, Rbl1, Hsp90aa1, Cks1b, Myh10, Kras, Chek1, Cdc14b, Slbp |
| CASEIN KINASE 2 | 8.39E-03 | 11 | Nolc1, Npm1, Topbp1, Csn3, Bdp1, Hsp90aa1, Mre11a, Srpk1, Slk, Slbp, Hmga1 |

**(C)** miR-322-5p

| **Pathway** | **P-value** | **# Genes (observed)** | **List of observed genes** |
| --- | --- | --- | --- |
| CYCLIN A2 | 3.52E-07 | 10 | Ccnd1, Ccna2, Cdc25a, Ckap2, Cdk1, Tbp, Chek1, Cdc6, Wee1, Hist1h3f |
| CYCLIN B1 | 1.11E-06 | 9 | Ccnd1, Ccna2, Aurka, Cdc25a, Bcl2, Cdk1, Chek1, Wee1, Hist1h3f |
| CYCLIN D1 | 1.26E-06 | 14 | Nras, Ccnd1, Ccna2, Cdc25a, Rbl1, Fosl1, Bcl2, Eif2s1, Tbp, Kras, Plaur, Ccne1, Cdc6, Hist1h3f |
| CYCLIN DEPENDENT KINASE | 6.62E-06 | 18 | Ppm1a, Id2, Smurf1, Ccnd1, Ccna2, Cdc7, E2f3, Parvb, Cdc25a, Rbl1, Pold3, Cdk8, Cdk1, Kras, Chek1, Cdc14b, Cdc6, Wee1 |
| CELL DIVISION CYCLE 2, G1 TO S AND G2 TO M | 1.04E-05 | 13 | Hmmr, Ccna2, Aurka, Cdc25a, Bcl2, Kif2c, Cdk1, Chek1, Cdc14b, Cdc6, Hmga1, Wee1, Hist1h3f |
| NIMA (NEVER IN MITOSIS GENE A) RELATED KINASE | 1.07E-05 | 7 | Cdc25a, Sgk1, Nek2, Hmga2, Cdk1, Vav2, Chek1 |
| POLO LIKE KINASE 1 | 1.77E-05 | 11 | Ccna2, Aurka, Clspn, Kif2c, Nek2, Cdk1, Slk, Chek1, Cdc6, Wee1, Hist1h3f |
| CELL DIVISION CYCLE 25C | 9.80E-05 | 6 | Ccna2, Cdc25a, Cdk1, Chek1, Wee1, Hist1h3f |
| CYCLIN E | 1.18E-04 | 7 | Ccnd1, Ccna2, Cdc25a, Cdk1, Chek1, Ccne1, Cdc6 |
| CYCLIN DEPENDENT KINASE INHIBITOR 1 | 2.39E-04 | 10 | Ccnd1, Ccna2, Cdc25a, Bcl2, Strap, Cdk1, Kras, Chek1, Cdc6, Wee1 |
| MINICHROMOSOME MAINTENANCE COMPLEX | 2.54E-04 | 5 | Ccnd1, Ccna2, Cdc7, Cdk1, Chek1 |
| ATAXIA TELANGIECTASIA AND RAD3 RELATED | 6.83E-04 | 7 | Aatf, Cdc7, Mcph1, Cdc25a, Clspn, Chek1, Cdc6 |
| PROTOONCOGENE / PROTEIN KINASE PIM | 1.10E-03 | 6 | Myb, Cdc25a, Fosl1, Bcl2, Kras, Hist1h3f |
| AURORA KINASE | 1.36E-03 | 7 | Kpnb1, Aurka, Mapre1, Kif2c, Cdk1, Kif2a, Hist1h3f |
| WEE1 HOMOLOG | 1.53E-03 | 4 | Ccna2, Cdk1, Chek1, Wee1 |
| E2F TRANSCRIPTION FACTOR 1 | 2.10E-03 | 6 | Ccnd1, Ccna2, E2f3, Myb, Cdk1, Chek1 |
| PROTEIN PHOSPHATASE 2 | 2.65E-03 | 11 | Ppm1a, Ckap2, Rbl1, Mapre1, Bcl2, Kif2c, Trip10, Eif4e, Ppp2r2a, Ppp2r1b, Cdc6 |
| BREAST CANCER 1, EARLY ONSET | 3.37E-03 | 6 | Aurka, Mcph1, Cdc25a, Rad51, Clspn, Chek1 |
| CELL DIVISION CYCLE 42 | 3.50E-03 | 8 | Ppm1a, Baiap2, Nras, Parvb, Fnbp1l, Trip10, Vav2, Sh3bp1 |
| TYROSINE PROTEIN KINASE SRC | 7.00E-03 | 14 | Rap1b, Prkcd, E2f3, G6pdx, Stub1, Blk, Asap1, Trip10, Selp, Vav2, Slk, Csk, Khdrbs1, Abl1 |
| RIBOSOMAL PROTEIN S6 KINASE | 7.56E-03 | 7 | Fkbp1a, Apln, Pdcd6, Sgk1, Eif4e, Prkaa1, Nfya |
| WISKOTT ALDRICH SYNDROME LIKE | 8.92E-03 | 3 | Actr2, Snx9, Fnbp1l |
| SMALL GTP BINDING PROTEIN RAC | 9.70E-03 | 8 | Baiap2, Nras, Ccnd1, Alox5, Axl, Vav2, Plaur, Abl1 |

**(D)** miR-335-3p

| **Pathway** | **P-value** | **# Genes (observed)** | **List of observed genes** |
| --- | --- | --- | --- |
| NUCLEAR RECEPTOR SUBFAMILY 2, GROUP F, MEMBER 1 | 1.30E-03 | 2 | Nr2f1, Nr2f2 |
| CELL DIVISION CYCLE 2, G1 TO S AND G2 TO M | 3.63E-03 | 11 | Ccna2, Prc1, Espl1, Elavl1, Birc5, Fbxo5, Kif23, Kif11, Ncl, Ccnb2, Wee1 |
| CYCLIN B1 | 4.14E-03 | 6 | Ccna2, Prc1, Espl1, Birc5, Ccnb2, Wee1 |
| FIBROBLAST GROWTH FACTOR | 4.53E-03 | 18 | Dlx5, Lhx2, Inhba, Spred2, Dlx2, Nars, Odc1, Fgfrl1, Sox9, Grb14, Twist1, Spred1, Spry4, Epha4, Gja1, Fgf7, Ugdh, Zfp259 |
| POLO LIKE KINASE 1 | 4.95E-03 | 9 | Ccna2, Prc1, Espl1, Nek2, Fbxo5, Csnk2a2, Kif11, Gtse1, Wee1 |
| TGF BETA | 5.14E-03 | 29 | Skil, Dlx5, Lhx2, Mtmr4, Peli1, Timp3, Inhba, Pmepa1, Scgb3a2, Has2, Vcan, Dlx2, Tbx20, Chsy1, Hivep3, Sox9, Nlk, Tnc, Twist1, Twsg1, Fbln2, Id1, Fut8, Sema7a, Dlx3, Hmga2, Klf10, Cse1l, Wwp1 |
| HEPATOCYTE GROWTH FACTOR RECEPTOR | 5.82E-03 | 8 | Skil, Ezr, Elavl1, Odc1, Ptgs2, Nras, Hgf, Fgf7 |
| MINICHROMOSOME MAINTENANCE COMPLEX | 8.57E-03 | 4 | Ccna2, Dbf4, Mcm6, Mcm4 |
| HYPOXIA INDUCIBLE FACTOR 1, ALPHA SUBUNIT (BASIC HELIX LOOP HELIX TRANSCRIPTION FACTOR) | 9.78E-03 | 9 | Abcb1a, Twist1, Ptgs2, Cxcl12, Id1, Arnt2, Abcc1, Rbms1, Egln1 |

**(E)** miR-335-5p

| **Pathway** | **P-value** | **# Genes (observed)** | **List of observed genes** |
| --- | --- | --- | --- |
| CYCLIN DEPENDENT KINASE INHIBITOR 1 | 7.54E-03 | 6 | Ccne2, Ccna2, Ppm1g, Trim28, Bcl2, Bmi1 |
| CYCLIN A2 | 9.97E-03 | 4 | Ccne2, Ccna2, Trim28, Plk1 |

**(F)** miR-351-5p

| **Pathway** | **P-value** | **# Genes (observed)** | **List of observed genes** |
| --- | --- | --- | --- |
| MATRIX METALLOPROTEINASE | 1.42E-03 | 9 | Vegfa, Mapk12, Ptk2, Cxcl12, Adam17, Ctgf, Nov, Dpp4, Mmp12 |
| THROMBOSPONDIN 1 | 1.53E-03 | 4 | Vegfa, Cdh5, Ptk2, Ctgf |
| CADHERIN 5, TYPE 2 (VASCULAR ENDOTHELIUM) | 2.60E-03 | 4 | Vegfa, Cdh5, Ptk2, Src |

**(G)** miR-503-5p

| **Pathway** | **P-value** | **# Genes (observed)** | **List of observed genes** |
| --- | --- | --- | --- |
| CYCLIN A2 | 8.33E-06 | 5 | Chek1, Cdt1, Cdc6, Ccnd1, Wee1 |
| CYCLIN E | 1.16E-04 | 4 | Chek1, Ccne1, Cdc6, Ccnd1 |
| MINICHROMOSOME MAINTENANCE COMPLEX | 3.07E-04 | 3 | Chek1, Ccnd1, Emb |
| ATAXIA TELANGIECTASIA AND RAD3 RELATED | 3.42E-04 | 4 | Chek1, Cdc6, Obfc1, Brca1 |
| CELL DIVISION CYCLE 25C | 6.85E-04 | 3 | Chek1, Brca1, Wee1 |
| S PHASE KINASE ASSOCIATED PROTEIN 2 (P45) | 8.30E-04 | 3 | Cdt1, Ccnd1, Itgb1 |
| CYCLIN DEPENDENT KINASE | 1.24E-03 | 6 | Chek1, Cdt1, Smurf1, Cdc6, Ccnd1, Wee1 |
| MAP KINASE INTERACTING SERINE/THREONINE KINASE | 1.80E-03 | 2 | Eif4g1, Eif4e |
| CYCLIN B1 | 1.97E-03 | 3 | Chek1, Ccnd1, Wee1 |
| PROTOONCOGENE / PROTEIN KINASE PIM | 2.40E-03 | 3 | Pim3, Fosl1, Myb |
| CYCLIN DEPENDENT KINASE INHIBITOR 1 | 2.66E-03 | 4 | Chek1, Cdc6, Ccnd1, Wee1 |
| E2F TRANSCRIPTION FACTOR 1 | 3.41E-03 | 3 | Chek1, Ccnd1, Myb |
| CYCLIN D1 | 4.40E-03 | 4 | Ccne1, Cdc6, Ccnd1, Fosl1 |
| BREAST CANCER 2, EARLY ONSET | 4.88E-03 | 2 | Chek1, Brca1 |
| CELL DIVISION CYCLE 2, G1 TO S AND G2 TO M | 4.97E-03 | 4 | Kif23, Chek1, Cdc6, Wee1 |
| TUMOR PROTEIN P53 | 5.58E-03 | 6 | Bcl2l12, Eif4g1, Chek1, Obfc1, Brca1, Emb |
| WEE1 HOMOLOG | 6.02E-03 | 2 | Chek1, Wee1 |

**(H)** miR-133a-3p/miR-133b-3p

| **Pathway** | **P-value** | **# Genes (observed)** | **List of observed genes** |
| --- | --- | --- | --- |
| V AKT MURINE THYMOMA VIRAL ONCOGENE HOMOLOG 1 | 1.25E-03 | 11 | Fscn1, Ppp2cb, Dlx3, Arrb2, Prex1, Abcc1, Efna4, Faim, Cxcl12, Mcl1, Arhgdia |
| NUCLEAR FACTOR (ERYTHROID DERIVED 2) LIKE 2 | 1.90E-03 | 4 | Abcc1, Gclc, Mafg, Srxn1 |
| FMS LIKE RECEPTOR TYROSINE KINASE 3 | 2.09E-03 | 4 | Cebpa, Cxcl12, Dlx1, Mcl1 |
| SMALL GTP BINDING PROTEIN RAC | 4.24E-03 | 6 | Cttn, Fscn1, Limk1, Prex1, Cxcl12, Arhgdia |
| RIBOSOMAL PROTEIN S6 KINASE | 6.29E-03 | 5 | Ppp2cb, Fkbp1a, Gclc, Rps6ka1, Nfya |

**(I)** miR-155-5p

| **Pathway** | **P-value** | **# Genes (observed)** | **List of observed genes** |
| --- | --- | --- | --- |
| CALCINEURIN (PROTEIN PHOSPHATASE 3) | 3.70E-04 | 9 | Calm1, Myoz2, Mef2a, Kcnj2, Abcc9, Pln, Map3k8, Pink1, Gnaq |
