# Supplementary material for: Integrative Analysis of MicroRNA and mRNA Data Reveals an Orchestrated Function of MicroRNAs in Skeletal Myocyte Differentiation in Response to TNF-α or IGF1
Source: PLoS One. 2015 Aug 13;10(8):e0135284. doi: 10.1371/journal.pone.0135284 (PMC4536022; doi:10.1371/journal.pone.0135284)
Supplement: S7 Table — We showed results for genes targeted by at least three miRNAs and refer to these as collectively targeted genes. Collectively targeted genes were associated with (A) GO terms ‘biological processes’. (B) The top 20 enriched MeSH disease terms of collectively targeted genes. (DOCX) [file pone.0135284.s013.docx]

**S7 Table. Enrichment of collectively targeted genes in GO and disease terms.**

We showed results for genes targeted by at least three miRNAs and refer to these as collectively targeted genes. Collectively targeted genes were enriched for **(A)** GO terms ‘biological processes’. **(B)** The top 20 enriched MeSH disease terms of collectively targeted genes.

**(A)**

| **GO-Term** | **P-value** | **# Genes (observed)** | **# Genes (expected)** | **# Genes (total)** |
| --- | --- | --- | --- | --- |
| cellular process | 1.51E-07 | 70 | 47.154158 | 13009 |
| primary metabolic process | 6.85E-07 | 50 | 27.841578 | 7681 |
| cellular metabolic process | 1.13E-06 | 49 | 27.352239 | 7546 |
| metabolic process | 5.79E-06 | 54 | 33.496162 | 9241 |
| mRNA splice site selection | 7.43E-06 | 3 | 0.0398721 | 11 |
| positive regulation of cell migration | 1.50E-05 | 7 | 0.7938166 | 219 |
| positive regulation of cell motility | 1.68E-05 | 7 | 0.8083156 | 223 |
| nervous system development | 1.83E-05 | 16 | 4.7846482 | 1320 |
| regulation of neurogenesis | 1.92E-05 | 9 | 1.5006397 | 414 |
| positive regulation of cellular component movement | 2.05E-05 | 7 | 0.8336887 | 230 |
| positive regulation of locomotion | 2.23E-05 | 7 | 0.8445629 | 233 |
| cellular macromolecule metabolic process | 2.94E-05 | 37 | 19.805544 | 5464 |
| positive regulation of cell development | 3.17E-05 | 6 | 0.6017058 | 166 |
| cell-cell signaling | 3.75E-05 | 11 | 2.4901919 | 687 |
| establishment of localization | 3.78E-05 | 26 | 11.541151 | 3184 |
| regulation of nervous system development | 4.23E-05 | 9 | 1.6601279 | 458 |
| neuron projection morphogenesis | 4.82E-05 | 8 | 1.3012793 | 359 |
| spliceosomal complex assembly | 5.01E-05 | 3 | 0.0724947 | 20 |
| localization | 5.11E-05 | 29 | 13.951599 | 3849 |
| neuron development | 7.05E-05 | 10 | 2.2074627 | 609 |

**(B)**

| **MeSH-Term** | **P-value** | **# Genes (observed)** | **# Genes (expected)** | **# Genes (total)** |
| --- | --- | --- | --- | --- |
| Tendinopathy | 3.54E-06 | 7 | 0.64125271 | 142 |
| Pleural Neoplasms | 8.86E-06 | 14 | 3.51785819 | 779 |
| Neoplasms, Adipose Tissue | 9.26E-06 | 12 | 2.58759017 | 573 |
| Fibroadenoma | 1.63E-05 | 8 | 1.12445018 | 249 |
| Idiopathic Interstitial Pneumonias | 2.05E-05 | 9 | 1.52184623 | 337 |
| Paget Disease, Extramammary | 2.44E-05 | 6 | 0.57803061 | 128 |
| Neoplasms, Fibroepithelial | 2.64E-05 | 9 | 1.57152073 | 348 |
| Pleural Diseases | 3.24E-05 | 16 | 5.05325201 | 1119 |
| Lymphangiomyoma | 3.43E-05 | 6 | 0.61415753 | 136 |
| Adenocarcinoma, Papillary | 3.54E-05 | 10 | 2.04568647 | 453 |
| Lymphatic Vessel Tumors | 3.63E-05 | 7 | 0.91672043 | 203 |
| Retinal Neoplasms | 4.34E-05 | 12 | 3.025629 | 670 |
| Leukopenia | 5.21E-05 | 16 | 5.2564659 | 1164 |
| Neoplastic Processes | 5.43E-05 | 48 | 30.6943288 | 6797 |
| Bartonella Infections | 5.66E-05 | 5 | 0.41094364 | 91 |
| Bartonellaceae Infections | 5.66E-05 | 5 | 0.41094364 | 91 |
| Liposarcoma | 6.14E-05 | 9 | 1.7521553 | 388 |
| Histiocytoma, Malignant Fibrous | 6.88E-05 | 6 | 0.69544308 | 154 |
| Histiocytoma | 6.91E-05 | 9 | 1.77925048 | 394 |
| Idiopathic Pulmonary Fibrosis | 7.38E-05 | 8 | 1.39088617 | 308 |
