# Supplementary material for: Integrative Analysis of MicroRNA and mRNA Data Reveals an Orchestrated Function of MicroRNAs in Skeletal Myocyte Differentiation in Response to TNF-α or IGF1
Source: PLoS One. 2015 Aug 13;10(8):e0135284. doi: 10.1371/journal.pone.0135284 (PMC4536022; doi:10.1371/journal.pone.0135284)
Supplement: S8 Table — Table of miRNAs showing the selected miRNA-mRNA relations of high interest based on the selection workflow depicted in Fig 1. Column “predicted to be targeted by….” is based on data of this study whereas column “previous evidence….” is based on literature search. (DOCX) [file pone.0135284.s014.docx]

**S8 Table. MiRNA-mRNA relations of high interest.**

Table of miRNAs showing the selected miRNA-mRNA relations of high interest based on the selection workflow depicted in Fig. 1. Column “predicted to be targeted by….” is based on data of this study whereas column “previous evidence….” is based on literature search.

| gene | predicted to be targeted by miR- | previous evidence |
| --- | --- | --- |
| Bard1 | 335-3p | no |
| Ccnd1 | 322-5p; 503; 206-3p | yes; yes; yes |
| Chek1 | 335-5p; 335-3p; 503; 206-3p | no; no; no; no |
| Clspn | 322-5p | no |
| Cxcl12 | 335-5p; 335-3p; 133b-3p; 351-5p | no; no; no; no |
| Fbxo5 | 335-3p | no |
| Fgf7 | 335-3p | no |
| Gja1 | 322-3p; 335-3p | no; no |
| Hmga2 | 322-5p | no |
| Id2 | 322-5p | no |
| Mcm10 | 206-3p | no |
| Mybl2 | 335-3p | no |
| Nr4a2 | 206-3p | yes |
| Plaur | 335-3p | no |
| Rrm2 | 335-5p | no |
| Selp | 322-5p | no |
| Serpinb2 | 335-3p | no |
| Serpine1 | 322-3p | no |
| Unc5b | 335-3p | no |
| Vegfa | 322-5p; 351-5p; 206-3p | no; no; yes |
| Ctbp2 | 322-3p; 133a-3p; 351-5p; 206-3p; 133b-3p | no; no;no; no; no |
